# Supplementary material for: A protein-independent fluorescent RNA aptamer reporter system for plant genetic engineering
Source: Nat Commun. 2020 Jul 31;11:3847. doi: 10.1038/s41467-020-17497-7 (PMC7395781; doi:10.1038/s41467-020-17497-7)
Supplement: Supplementary file 1 — Supplementary Information [file 41467_2020_17497_MOESM1_ESM.pdf]

Supplementary Information

A protein-independent fluorescent RNA aptamer reporter system for plant genetic engineering

Bai et al.

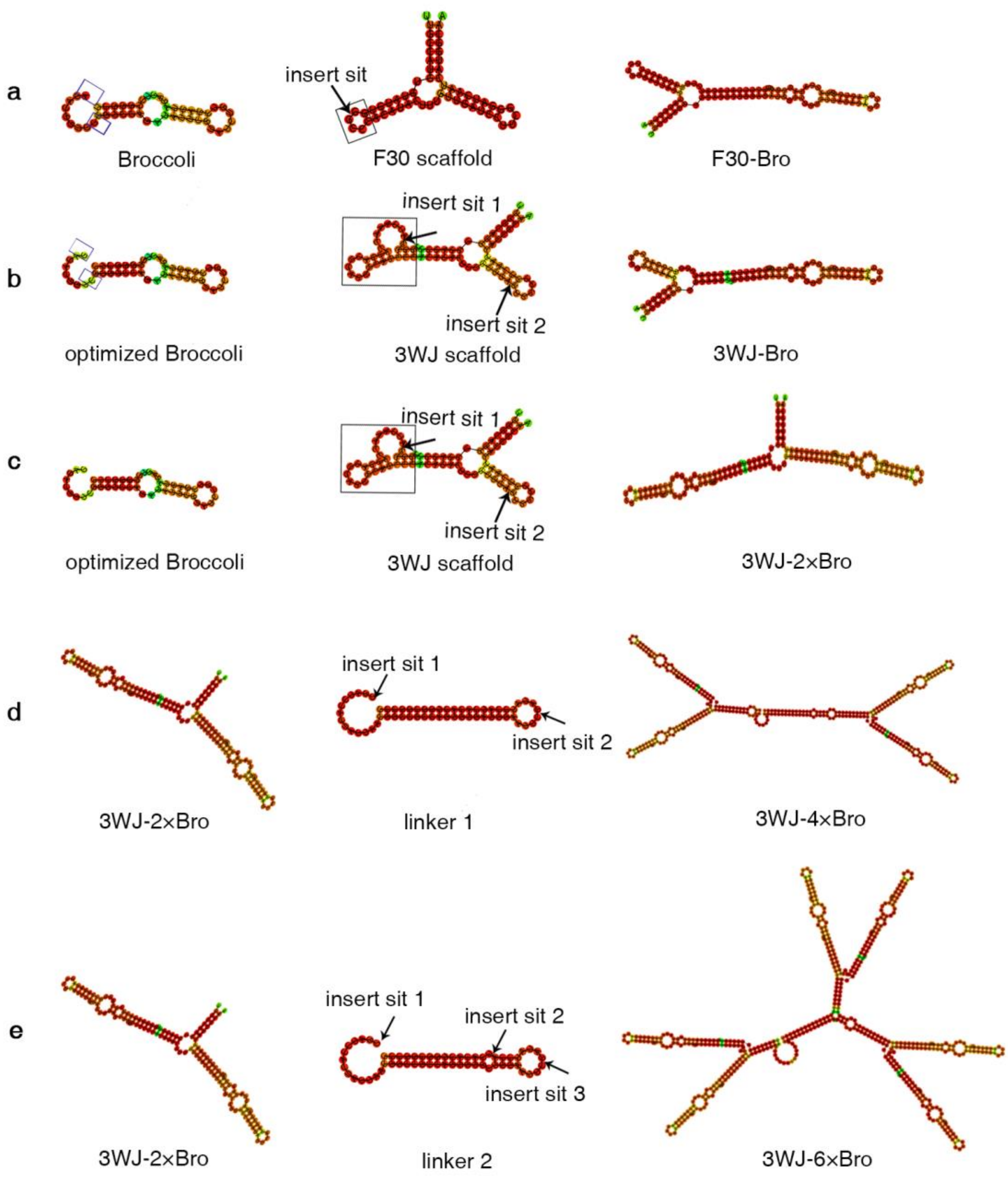

Supplementary Figure 1 | Designations and minimum free energy (MFE) secondary structure predictions of the 3WJ-Bro aptamer series. The structures are colored by base-pairing probabilities in the MFE structure drawing, from 0 to 1 (the redder, the higher). Black frame and black solid arrow indicate the modification site and insertion site, respectively.

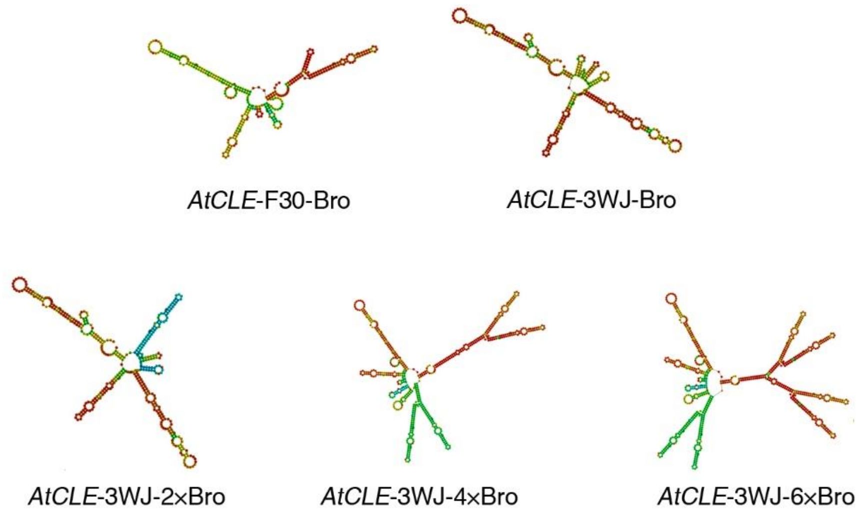

Supplementary Figure 2 | MFE secondary structure predictions of different aptamers after tagging to *AtCLE* mRNA. The predicted conformations are based on calculations performed using the RNAfold webserver. The structures are colored by base-pairing probabilities in the MFE structure drawing, from 0 to 1 (the redder, the higher).

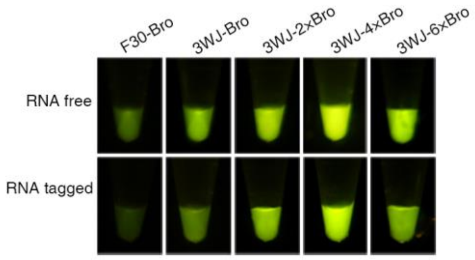

Supplementary Figure 3 | Fluorescence imaging of aptamers after tagging to *AtCLE* mRNA *in vitro*. 200 nM RNA in 50 μL 1× PEPES buffer (PH 7.2) supplemented with 10 μM DFHBI-1T. Three independent experiments showed similar results.

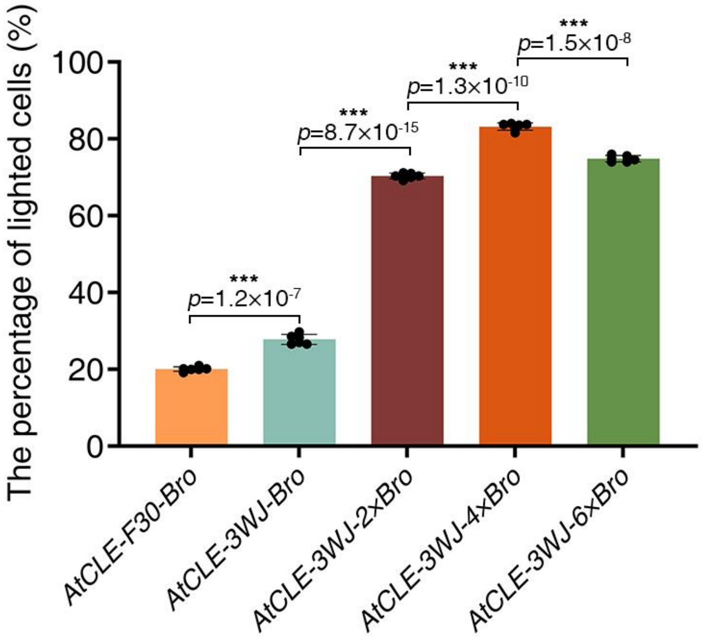

Supplementary Figure 4 | Percentage of fluorescent *E. coli* cells to total cells expressing *AtCLE* mRNAs tagged with different aptamers. Error bars depict the mean ± standard deviation (s.d.) and *p*-values are calculated using an unpaired, two-tailed, Student's *t*-test. ***p < 0.001. Experiments were repeated six independent times. Source Data are provided as a Source Data file.

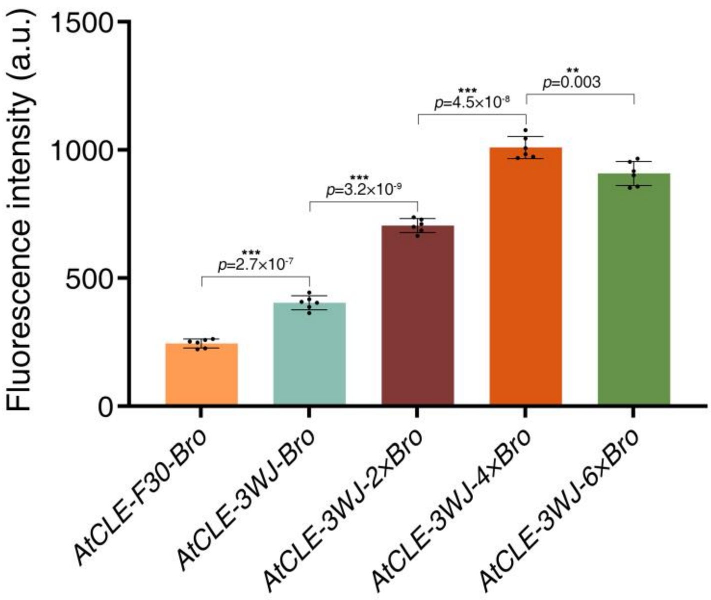

Supplementary Figure 5 | Fluorescence intensities of *E. coli* cells expressing *AtCLE* mRNAs tagged with different aptamers. Error bars depict the mean ± s.d. (n = 6) and *p*-values are calculated using an unpaired, two-tailed, Student's *t*-test. **p < 0.01, ***p < 0.001. Experiments were repeated three independent times. Source Data are provided as a Source Data file.

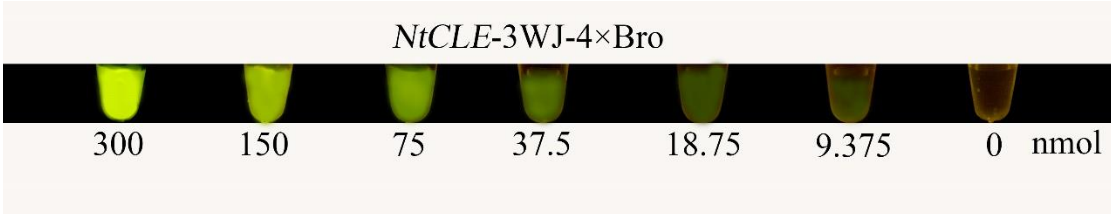

Supplementary Figure 6 | Representative imaging of fusion RNA *AtCLE-3WJ-4×Bro* after a serial dilution. Three repeated experiments showed the same results.

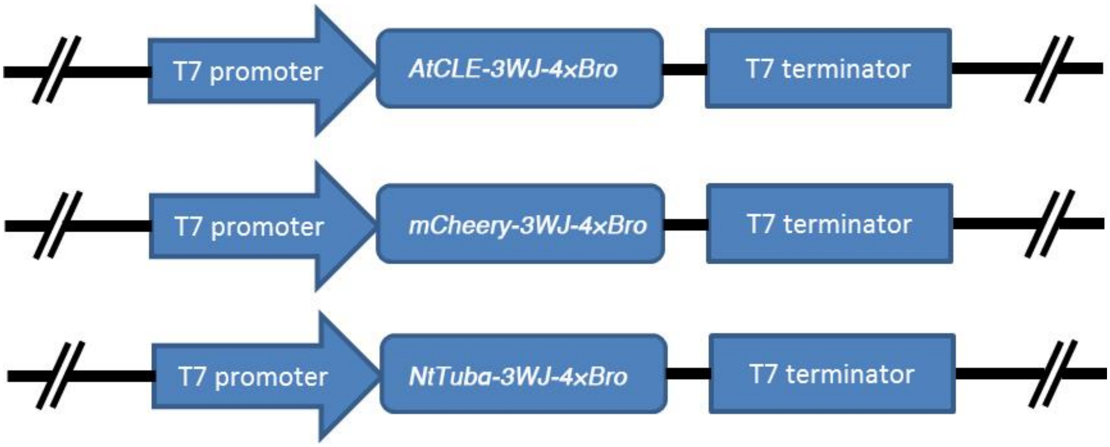

Supplementary Figure 7 | Schematics of the constructs used for expression of 3WJ-4×Bro-tagged mRNA in *E. coli* BL21 cells.

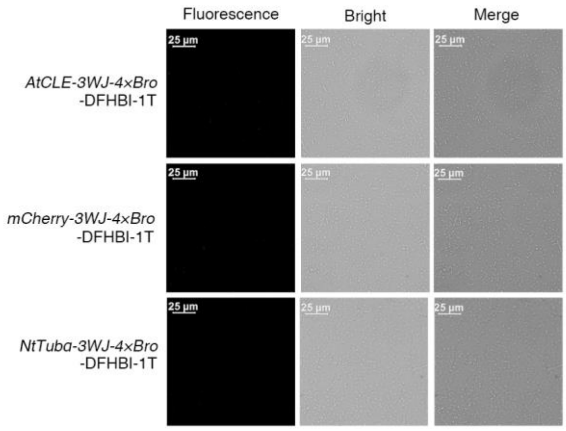

Supplementary Figure 8 | Representative images of *E. coli* cells expressing 3WJ-4×Bro-tagged mRNAs without DFHBI-1T incubation (control group). Scale bars, 25 μm. Experiments were conducted in triplicate with the same results.

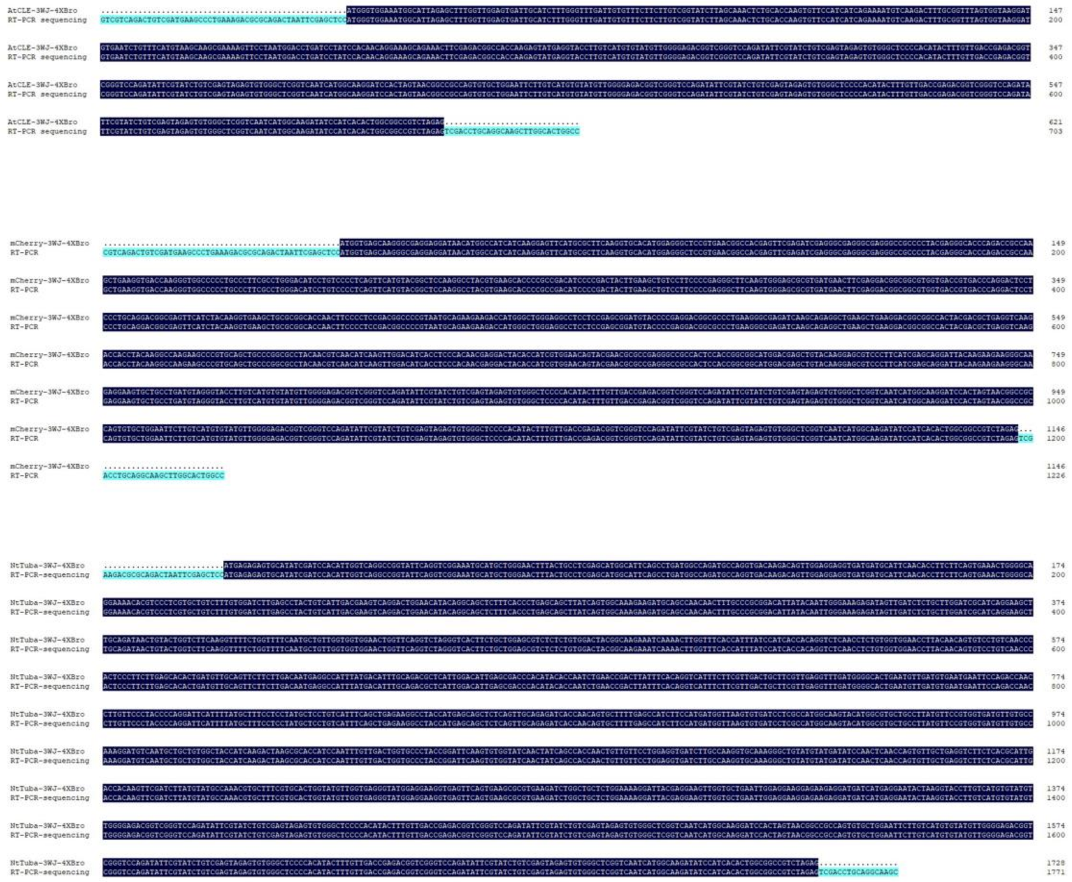

Supplementary Figure 9 | RT-PCR sequencing of 3WJ-4×Bro-tagged mRNAs in *E. coli* cells. The alignment was conducted by software DNAMAN 8.0 and the black indicated 100% identity between two sequences.

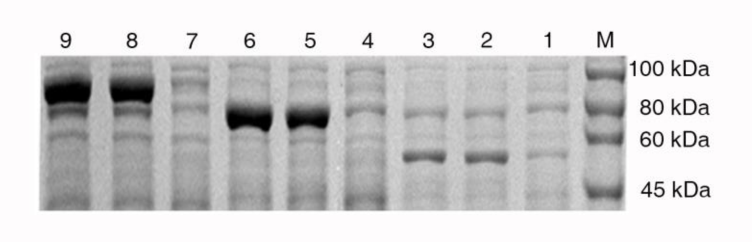

Supplementary Figure 10 | Prokaryotic expression of 3WJ-4×Bro-tagged genes. Total protein was extracted from *E. coli* strain Rosetta (DE3) after IPTG induction. The protein expression level and purity were estimated using a 10% tricine SDS-PAGE gel and Coomassie blue staining. Lane M, protein marker; Lane 1, total proteins from *E. coli* cells without inducible expression of *AtCLE*; Lane 2, total proteins from *E. coli* cells expressing *AtCLE*; Lane 3, total proteins from *E. coli* cells expressing *AtCLE-3WJ-4×Bro*; Lane 4, total proteins from *E. coli* cells without inducible expression of *mCherry*; Lane 5, total proteins from *E. coli* cells expressing *mCherry*; Lane 6, total proteins from *E. coli* cells expressing *mCherry -3WJ-4×Bro*; Lane 7, total proteins from *E. coli* cells without inducible expression of *NtTubα*; Lane 8, total proteins from *E. coli* cells expressing *NtTub*α; Lane 9, total proteins from *E. coli* cells expressing *NtTubα-3WJ-4×Bro*. Experiments were conducted in triplicate with the same results. Source Data are provided as a Source Data file.

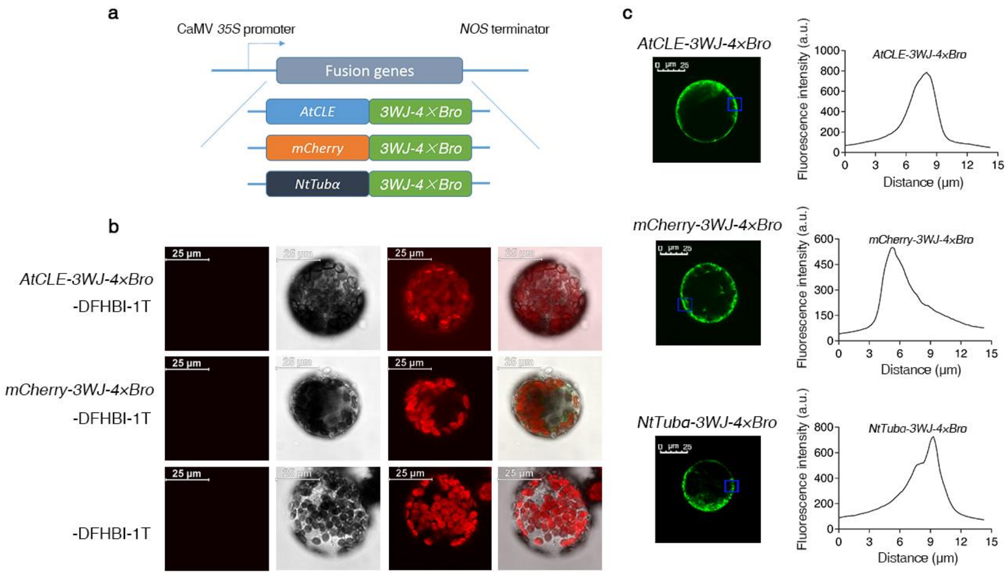

Supplementary Figure 11 | Imaging of 3WJ-4×Bro-tagged mRNAs in protoplasts.

(a) Schematics of constructs.

(b) Representative image of a protoplast before incubation with DFHBI-1T. c, Fluorescence signal and background measurement of mRNAs tagged with 3WJ-4×Bro in protoplasts. Blue lines (left) were used for the linescan (right). Six independent trials were performed with similar results. Scale bars, 25 μm.

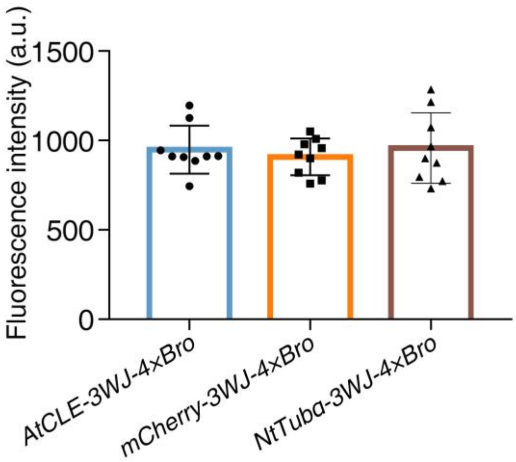

Supplementary Figure 12 | Quantification of fluorescence intensity in protoplasts expressing mRNAs tagged with 3WJ-4×Bro. Error bars depict the mean ± s.d. (n = 9). Experiments were repeated three independent times. Source Data are provided as a Source Data file.

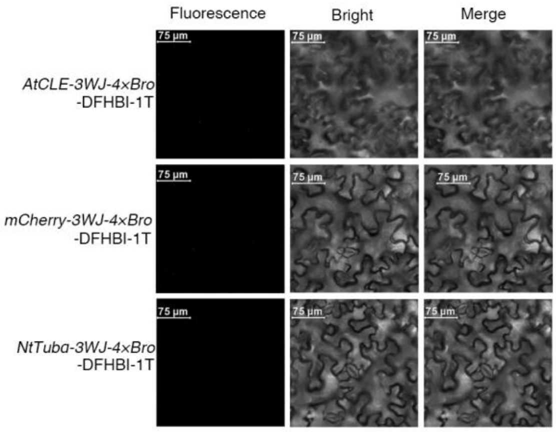

Supplementary Figure 13 | Representative confocal images of *Nicotiana benthamiana* leaves expressing three 3WJ-4×Bro-tagged mRNAs without DFHBI-1T incubation. Scale bars, 25 µm Experiments were conducted in triplicate with the same results.

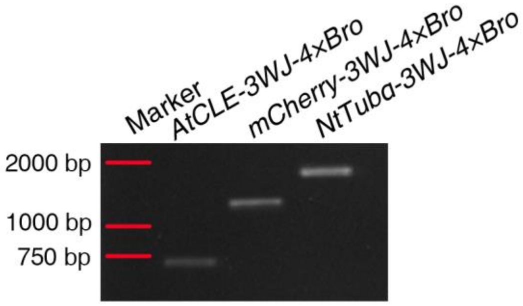

Supplementary Figure 14 | Integrity detection of mRNAs tagged with 3WJ-4×Bro in *Nicotiana benthamiana* cells. Total cellular RNAs were extracted and separated by urea denaturing gel. After washing, the gel image was stained with DFHBI-1T and photographed. Three independent trials were performed with the same results. Red lines indicate the band locations of markers on the gel. Source Data are provided as a Source Data file.

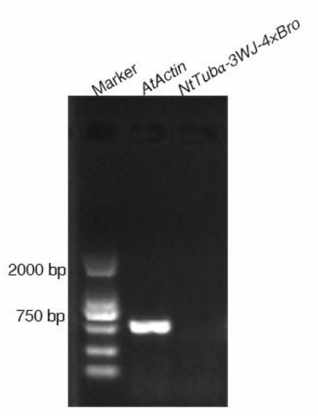

Supplementary Figure 15 | RT-PCR detection of *NtTubα-3WJ-4×Bro* mRNA in the *Arabidopsis thaliana* T1 transgenic line N-F1. *AtActin* was used as reference gene. Experiments were conducted in triplicate with the same results.

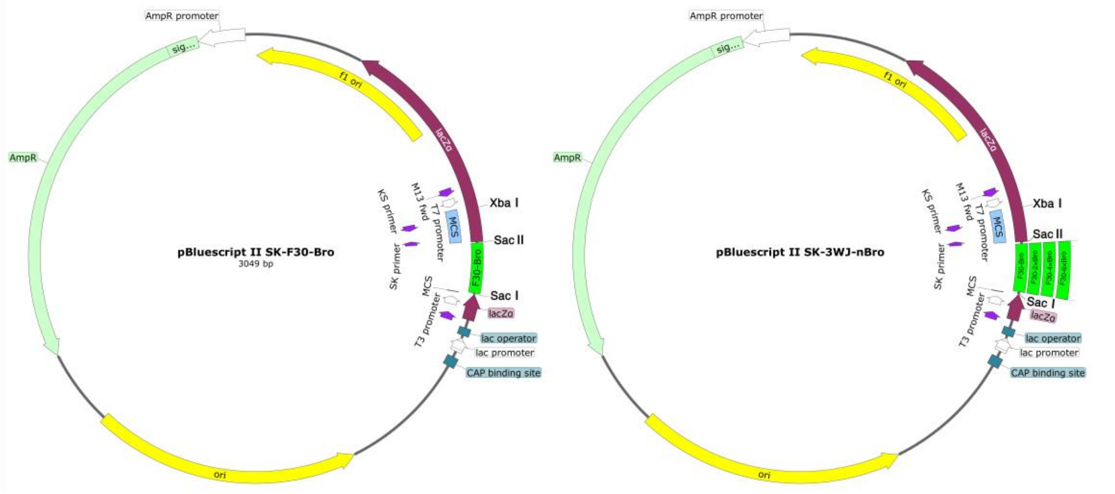

Supplementary Figure 16 | The constructs of pBluescript II SK-F30-Bro and pBluescript II SK-3WJ-nBro. 3WJ-nBro includes 3WJ-Bro, 3WJ-2×Bro, 3WJ-4×Bro and 3WJ-6×Bro.

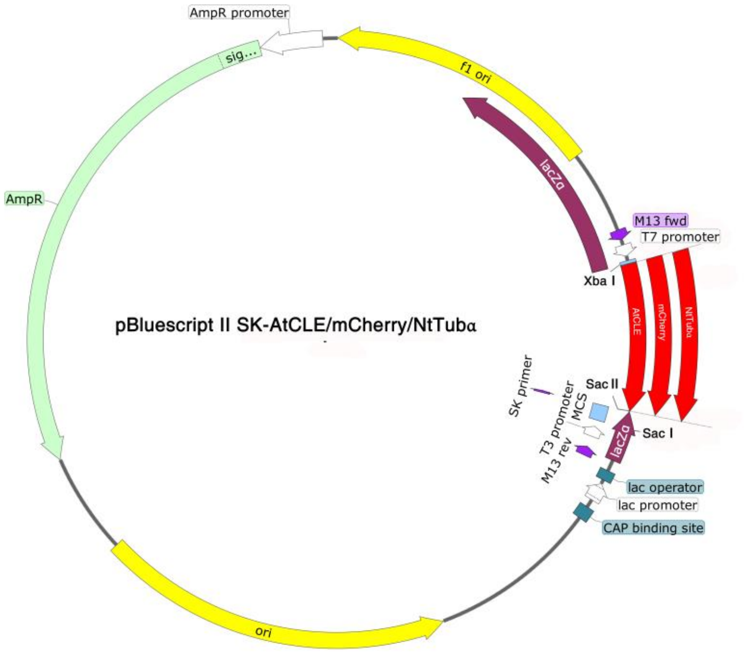

Supplementary Figure 17 | The constructs of pBluescript II SK-AtCLE, pBluescript II SKmCherry and pBluescript II SK-NtTubα.

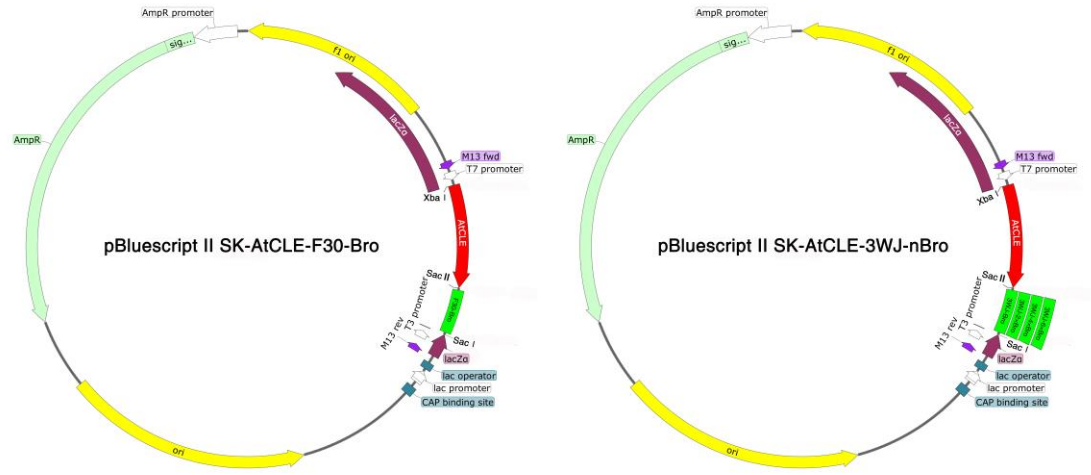

Supplementary Figure 18 | The constructs of pBluescript II SK-AtCLE-F30-Bro and pBluescript II SK-AtCLE-3WJ-nBro. 3WJ-nBro includes 3WJ-Bro, 3WJ-2×Bro, 3WJ-4×Bro and 3WJ-6×Bro.

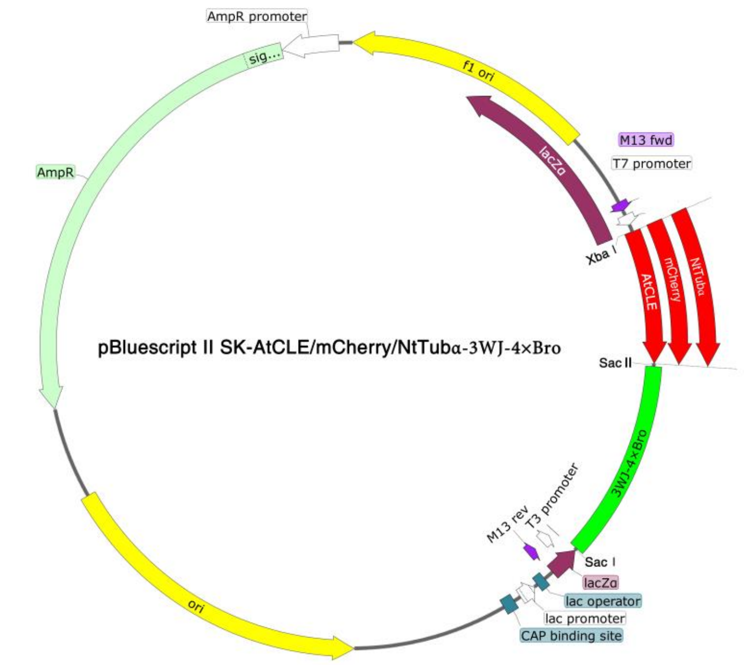

Supplementary Figure 19 | The constructs of pBluescript II SK-AtCL-3WJ-4×Bro, pBluescript II SK-mCherry-3WJ-4×Bro, pBluescript II SK-NtTubα-3WJ-4×Bro.

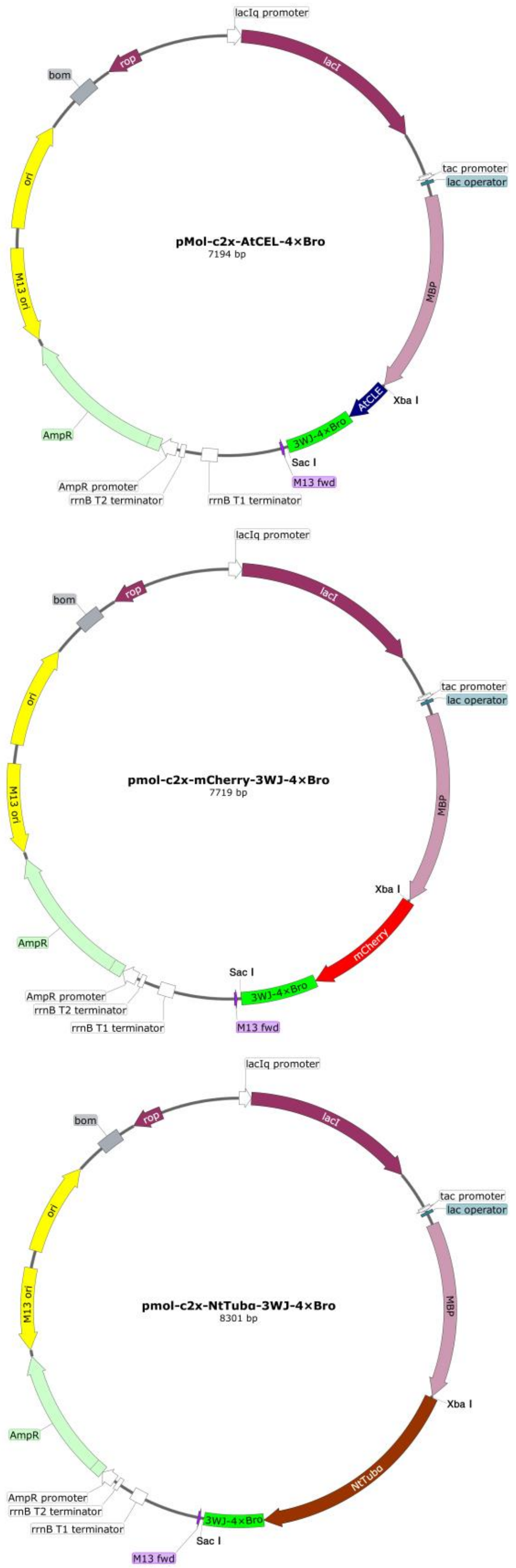

Supplementary Figure 20 | The constructs of recombinant pMol-c2x plasmids for expressing *AtCLE-3WJ-4×Bro*, *mCherry-3WJ-4×Bro* and *NtTubα-3WJ-4×Bro* in *E. coli* cells.

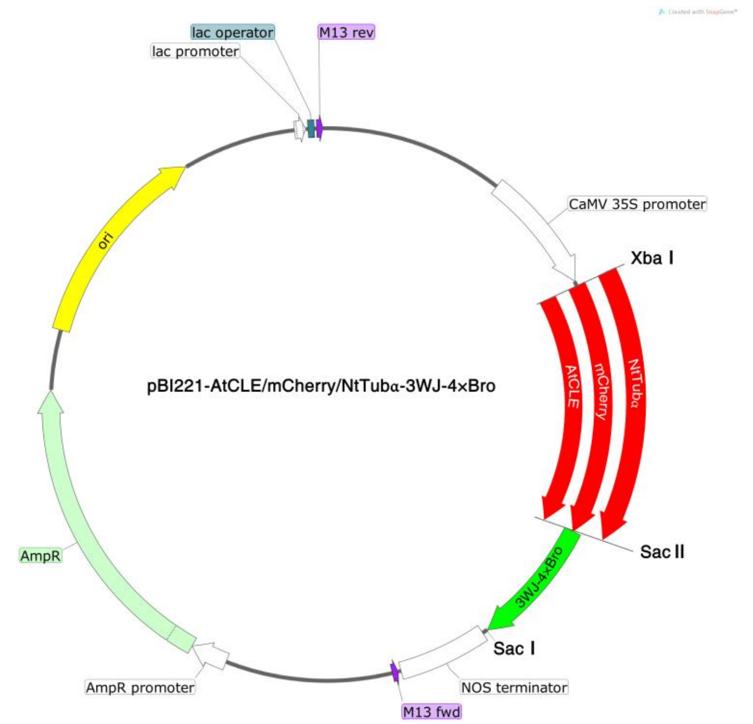

Supplementary Figure 21 | The constructs of recombinant Pbi221 for expressing *AtCLE-3WJ-4×Bro*, *mCherry-3WJ-4×Bro* and *NtTubα-3WJ-4×Bro* in *N. benthamiana* protoplasts.

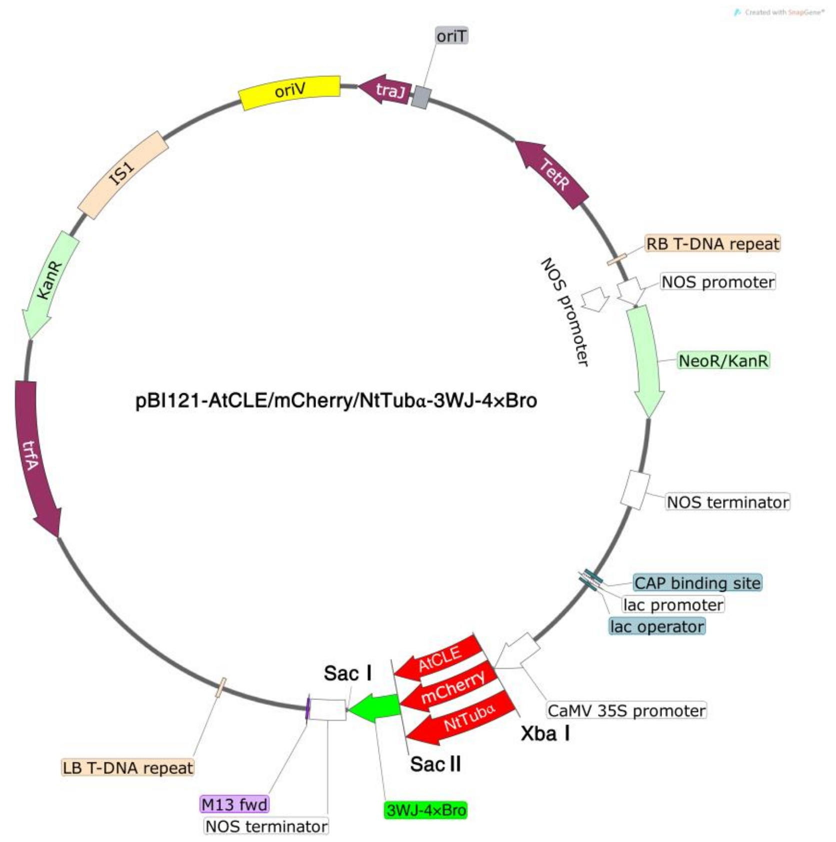

Supplementary Figure 22 | The constructs of recombinant Pbi121 for expressing *AtCLE-3WJ-4×Bro*, *mCherry-3WJ-4×Bro* and *NtTubα-3WJ-4×Bro* in *N. benthamiana* leaves.

Supplementary Table 1. List of sequences used in this study

Tested RNAs	DNA Sequence	Length (bp)
3WJ	TTGTCATGTGTATGTTGGGGATTAAACCCTGATTGAGTTCAGCCCACATACTTTG<br>TTGATTGGTTGTCAATCATGGCAA	79
Broccoli	GAGACGGTCGGGTCCAGATATTCGTATCTGTCGAGTAGAGTGTGGGCTC	49
3WJ-Bro	TTGTCATGTGTATGTTGGGGAGACGGTCGGGTCCAGATATTCGTATCTGTCGAGT<br>AGAGTGTGGGCTCCCCACATACTTTGTTGATTGGTTGTCAATCATGGCAA	105
3WJ-2×Bro	TTGTCATGTGTATGTTGGGGAGACGGTCGGGTCCAGATATTCGTATCTGTCGAGT<br>AGAGTGTGGGCTCCCCACATACTTTGTTGACCGAGACGGTCGGGTCCAGATATT<br>CGTATCTGTCGAGTAGAGTGTGGGCTCGGTCAATCATGGCAA	151
3WJ-4×Bro	GGTACCTTGTCATGTGTATGTTGGGGAGACGGTCGGGTCCAGATATTCGTATCTG<br>TCGAGTAGAGTGTGGGCTCCCCACATACTTTGTTGACCGAGACGGTCGGGTCCA<br>GATATTCGTATCTGTCGAGTAGAGTGTGGGCTCGGTCAATCATGGCAAGGATCCA<br>CTAGTAACGGCCGCCAGTGTGCTGGAATTCTTGTCATGTGTATGTTGGGGAGAC<br>GGTCGGGTCCAGATATTCGTATCTGTCGAGTAGAGTGTGGGCTCCCCACATACTT<br>TGTTGACCGAGACGGTCGGGTCCAGATATTCGTATCTGTCGAGTAGAGTGTGGG<br>CTCGGTCAATCATGGCAAGATATCCATCACACTGGCGGCCG	368
3WJ-6×Bro	GGTACCTTGTCATGTGTATGTTGGGGAGACGGTCGGGTCCAGATATTCGTATCTG<br>TCGAGTAGAGTGTGGGCTCCCCACATACTTTGTTGACCGAGACGGTCGGGTCCA<br>GATATTCGTATCTGTCGAGTAGAGTGTGGGCTCGGTCAATCATGGCAAGGATCCA<br>CTAGTAACGGCCGCCAGTGTGCTGGAATTCTTGTCATGTGTATGTTGGGGAGAC<br>GGTCGGGTCCAGATATTCGTATCTGTCGAGTAGAGTGTGGGCTCCCCACATACTT<br>TGTTGACCGAGACGGTCGGGTCCAGATATTCGTATCTGTCGAGTAGAGTGTGGG<br>CTCGGTCAATCATGGCAAGATATCCATTGTCATGTGTATGTTGGGGAGACGGTCG<br>GGTCCAGATATTCGTATCTGTCGAGTAGAGTGTGGGCTCCCCACATACTTTGTTG<br>ACCGAGACGGTCGGGTCCAGATATTCGTATCTGTCGAGTAGAGTGTGGGCTCGG<br>TCAATCATGGCAATCACACTGGCGGCCG	519
NtCEL-3WJ-4×Bro	ATGGGTGGAAATGGCATTAGAGCTTTGGTTGGAGTGATTGCATCTTTGGGTTTGA<br>TTGTGTTTCTTCTTGTCGGTATCTTAGCAAACTCTGCACCAAGTGTTCCATCATCA<br>GAAAATGTCAAGACTTTGCGGTTTAGTGGTAAGGATGTGAATCTGTTTCATGTAA<br>GCAAGCGAAAAGTTCCTAATGGACCTGATCCTATCCACAACAGGAAAGCAGAA<br>ACTTCGAGACGGCCACCAAGAGTATGACCGCGGGGTACCTTGTCATGTGTATGT<br>TGGGGAGACGGTCGGGTCCAGATATTCGTATCTGTCGAGTAGAGTGTGGGCTCC<br>CCACATACTTTGTTGACCGAGACGGTCGGGTCCAGATATTCGTATCTGTCGAGTA<br>GAGTGTGGGCTCGGTCAATCATGGCAAGGATCCACTAGTAACGGCCGCCAGTGT<br>GCTGGAATTCTTGTCATGTGTATGTTGGGGAGACGGTCGGGTCCAGATATTCGTA<br>TCTGTCGAGTAGAGTGTGGGCTCCCCACATACTTTGTTGACCGAGACGGTCGGG<br>TCCAGATATTCGTATCTGTCGAGTAGAGTGTGGGCTCGGTCAATCATGGCAAGAT<br>ATCCATCACACTGGCGGCCG	620
mCherry-3WJ-4×Bro	ATGGTGAGCAAGGGCGAGGAGGATAACATGGCCATCATCAAGGAGTTCATGCGC<br>TTCAAGGTGCACATGGAGGGCTCCGTGAACGGCCACGAGTTCGAGATCGAGGG<br>CGAGGGCGAGGGCCGCCCCTACGAGGGCACCCAGACCGCCAAGCTGAAGGTG<br>ACCAAGGGTGGCCCCCTGCCCTTCGCCTGGGACATCCTGTCCCCTCAGTTCATG	1145

	TACGGCTCCAAGGCCTACGTGAAGCACCCCGCCGACATCCCCGACTACTTGAAG<br>CTGTCCTTCCCCGAGGGCTTCAAGTGGGAGCGCGTGATGAACTTCGAGGACGG<br>CGGCGTGGTGACCGTGACCCAGGACTCCTCCCTGCAGGACGGCGAGTTCATCTA<br>CAAGGTGAAGCTGCGCGGCACCAACTTCCCCTCCGACGGCCCCGTAATGCAGA<br>AGAAGACCATGGGCTGGGAGGCCTCCTCCGAGCGGATGTACCCCGAGGACGGC<br>GCCCTGAAGGGCGAGATCAAGCAGAGGCTGAAGCTGAAGGACGGCGGCCACT<br>ACGACGCTGAGGTCAAGACCACCTACAAGGCCAAGAAGCCCGTGCAGCTGCCC<br>GGCGCCTACAACGTCAACATCAAGTTGGACATCACCTCCCACAACGAGGACTAC<br>ACCATCGTGGAACAGTACGAACGCGCCGAGGGCCGCCACTCCACCGGCGGCAT<br>GGACGAGCTGTACAAGGAGCGTCCCTTCATCGAGCAGGATTACAAGAAGAAGG<br>GCAAGAGGAAGTGCTGCCTGATGTAGCCGCGGGGTACCTTGTCATGTGTATGTT<br>GGGGAGACGGTCGGGTCCAGATATTCGTATCTGTCGAGTAGAGTGTGGGCTCCC<br>CACATACTTTGTTGACCGAGACGGTCGGGTCCAGATATTCGTATCTGTCGAGTAG<br>AGTGTGGGCTCGGTCAATCATGGCAAGGATCCACTAGTAACGGCCGCCAGTGTG<br>CTGGAATTCTTGTCATGTGTATGTTGGGGAGACGGTCGGGTCCAGATATTCGTAT<br>CTGTCGAGTAGAGTGTGGGCTCCCCACATACTTTGTTGACCGAGACGGTCGGGT<br>CCAGATATTCGTATCTGTCGAGTAGAGTGTGGGCTCGGTCAATCATGGCAAGATA<br>TCCATCACACTGGCGGCCG	
NtTubα-3WJ-4×Bro	ATGAGAGAGTGCATATCGATCCACATTGGTCAGGCCGGTATTCAGGTCGGAAAT<br>GCATGCTGGGAACTTTACTGCCTCGAGCATGGCATTCAGCCTGATGGCCAGATG<br>CCAGGTGACAAGACAGTTGGAGGAGGTGATGATGCATTCAACACCTTCTTCAGT<br>GAAACTGGGGCAGGAAAACACGTCCCTCGTGCTGTCTTTGTGGATCTTGAGCCT<br>ACTGTCATTGACGAAGTCAGGACTGGAACATACAGGCAGCTCTTTCACCCTGAG<br>CAGCTTATCAGTGGCAAAGAAGATGCAGCCAACAACTTTGCCCGCGGACATTAT<br>ACAATTGGGAAAGAGATAGTTGATCTCTGCTTGGATCGCATCAGGAAGCTTGCA<br>GATAACTGTACTGGTCTTCAAGGTTTTCTGGTTTTCAATGCTGTTGGTGGTGGAA<br>CTGGTTCAGGTCTAGGGTCACTTCTGCTGGAGCGTCTCTCTGTGGACTACGGCA<br>AGAAATCAAAACTTGGTTTCACCATTTATCCATCACCACAGGTCTCAACCTCTGT<br>GGTGGAACCTTACAACAGTGTCCTGTCAACCCACTCCCTTCTTGAGCACACTGA<br>TGTTGCAGTTCTTCTTGACAATGAGGCCATTTATGACATTTGCAGACGCTCATTG<br>GACATTGAGCGACCCACATACACCAATCTGAACCGACTTATTTCACAGGTCATTT<br>CTTCGTTGACTGCTTCGTTGAGGTTTGATGGGGCACTGAATGTTGATGTGAATGA<br>ATTCCAGACCAACCTTGTTCCCTACCCCAGGATTCATTTTATGCTTTCCTCCTATG<br>CTCCTGTCATTTCAGCTGAGAAGGCCTACCATGAGCAGCTCTCAGTTGCAGAGA<br>TCACCAACAGTGCTTTTGAGCCATCTTCCATGATGGTTAAGTGTGATCCTCGCCA<br>TGGCAAGTACATGGCGTGCTGCCTTATGTTCCGTGGTGATGTTGTGCCAAAGGAT<br>GTCAATGCTGCTGTGGCTACCATCAAGACTAAGCGCACCATCCAATTTGTTGACT<br>GGTGCCCTACCGGATTCAAGTGTGGTATCAACTATCAGCCACCAACTGTTGTTCC<br>TGGAGGTGATCTTGCCAAGGTGCAAAGGGCTGTATGTATGATATCCAACTCAAC<br>CAGTGTTGCTGAGGTCTTCTCACGCATTGACCACAAGTTCGATCTTATGTATGCC<br>AAACGTGCTTTCGTGCACTGGTATGTTGGTGAGGGTATGGAGGAAGGTGAGTTC<br>AGTGAAGCGCGTGAAGATCTGGCTGCTCTGGAAAAGGATTACGAGGAAGTTGG<br>TGCTGAATTGGAGGAAGGAGAAGAGGATGATCATGAGGAATACTAACCGCGGG<br>GTACCTTGTCATGTGTATGTTGGGGAGACGGTCGGGTCCAGATATTCGTATCTGT	1727

	CGAGTAGAGTGTGGGCTCCCCACATACTTTGTTGACCGAGACGGTCGGGTCCAG<br>ATATTCGTATCTGTCGAGTAGAGTGTGGGCTCGGTCAATCATGGCAAGGATCCAC<br>TAGTAACGGCCGCCAGTGTGCTGGAATTCTTGTCATGTGTATGTTGGGGAGACG<br>GTCGGGTCCAGATATTCGTATCTGTCGAGTAGAGTGTGGGCTCCCCACATACTTT<br>GTTGACCGAGACGGTCGGGTCCAGATATTCGTATCTGTCGAGTAGAGTGTGGGC<br>TCGGTCAATCATGGCAAGATATCCATCACACTGGCGGCCG	

Supplementary Table 2. List of primers used in this study

Role	Primer name	Sequences
In vitro transcription	M13 F	GTAAAACGACGGCCAGT
	M13 R	CAGGAAACAGCTATGAC
Clone of *AtCLE*	AtC F	GCTCTAGAATGGGTGGAAATGGC (*Xba*I)
	AtC R	GCCGCGGTCATACTCTTGGTGGCCGTC (*Sac*II)
Clone of *mCherry*	mCh F	GCTCTAGAATGGTGAGCAAGGGCG (*Xba*I)
	mCh R	GCCGCGGCTACATCAGGCAGCAC (*Sac*II)
Clone of *NtTubα*	NtT F	GCTCTAGAATGAGAGAGTGCATATC (*Xba*I)
	NtT R	GCCGCGGTTAGTATTCCTCATGATCAT (*Sac*II)
Construct of pMol-AtCLE-3WJ-4×Bro	pM-At-3WJ F	CGGAGCTCCATGGGTGGAAATGGC (*Sac*I)
	pM-At-3WJ R	GCTCTAGACGGCCGCCAGTGTGATG (*Xba*I)
Construct of pMol-AtCLE	pM-At F	CGGAGCTCCATGGGTGGAAATGGC (*Sac*I)
	pM-At R	GCTCTAGATCATACTCTTGGTGGCCGTC (*Xba*I)
Construct of pMol-mCherry-3WJ-4×Bro	pM-mC-3WJ F	CGGAGCTCCATGGTGAGCAAGGGCG (*Sac*I)
	pM-mC-3WJ R	GCTCTAGACGGCCGCCAGTGTGATG (*Xba*I)
Construct of pMol-mCherry	pM-mC F	CGGAGCTCCATGGTGAGCAAGGGCG (*Sac*I)
	pM-mC R	CCTCTAGACTACATCAGGCAGCAC (*Xba*I)
Construct of pMol- NtTubα-3WJ-4×Bro	pM-Nt-3WJ F	CGGAGCTCCATGAGAGAGTGCATATC (*Sac*I)
	pM-Nt-3WJ R	GCTCTAGACGGCCGCCAGTGTGATG(*Xba*I)
Construct of pMol- NtTubα	pM-Nt F	CGGAGCTCCATGAGAGAGTGCATATC (*Sac*I)
	pM-Nt R	CCTCTAGA TTAGTATTCCTCATGATCAT (*Xba*I)
TRV2 sequencing	TRV2 F	TCACTTACCCGAGTTAACGAG
	TRV2 R	TGTCTTCGGGACATGCCCC
Construct of TRV2-mCherry-3WJ-4×Bro	TRV2-mC F	TGCTCTAGAATGGTGAGCAAGGGC (*Xba*I)
	TRV2-mC R	CATGCCATGGCGGCCGCCAGTGTGATG (*Nco*I)
Construct of pBI121(pBI221)-AtCLE-3WJ-4×Bro	pB-At F	GCTCTAGAATGGGTGGAAATGGC (*Xba*I)
	pB-At R	CGGAGCTCCCGGCCGCCAGTGTGATG (*Sac*I)
Construct of pBI121(pBI221)-mCherry-3WJ-4×Bro	pB-mC F	GCTCTAGAATGGTGAGCAAGGGCG (*Xba*I)
	pB-mC R	CGGAGCTCCCGGCCGCCAGTGTGATG (*Sac*I)
Construct of pBI121(pBI221)-NtTubα-3WJ-4×Bro	pB-Nt F	GCTCTAGAATGAGAGAGTGCATATC (*Xba*I)
	pB-Nt F	CGGAGCTCCCGGCCGCCAGTGTGATG (*Sac*I)
Identification of transgenic plants	35S	TGAGACTTTTCAACAAAGGGT
	NOS	TTCCCGATCTAGTAACATAG
RT-PCR for mRNA of NtTubα-3WJ-4×Bro	RT F	AGTACGAACGCGCCGAGG
	RT R	GGGGAGCCCACACTCTAC
RT-PCR for mRNA of AtActinβ2 (reference gene)	Actin	AGGAAGTTGACGAGCAGAT
	Actin	AGACCAGTAGGTGGGATGT

Underlining represents recognition sites of restriction enzymes shown in brackets following the sequences.
