# Supplementary material for: A protein-independent fluorescent RNA aptamer reporter system for plant genetic engineering
Source: Nat Commun. 2020 Jul 31;11:3847. doi: 10.1038/s41467-020-17497-7 (PMC7395781; doi:10.1038/s41467-020-17497-7)
Supplement: Supplementary file 4 — Source Data [file 41467_2020_17497_MOESM4_ESM.zip › Source Data/Description of Source Data.docx]

**Source Data Underlying Fig. 1a.** Excitation (dashed) and emission (solid) spectra of RNA aptamers.

**Source Data Underlying Fig. 1b.** Representative fluorescence images of 3WJ-nBro series from a Blue Light Gel Imager.

**Source Data Underlying Fig. 1c.** Fluorescence quantification of 3WJ-nBro series.

**Source Data Underlying Fig. 1d.** Fluorescence detection within 5 h after DFHBI-1T administration.

**Source Data Underlying Fig. 1e.** Normalized fluorescence decay rate calculated as the ratio of remaining fluorescence to initial fluorescence.

**Source Data Underlying Fig. 2b.** Fluorescence changes of 3WJ-nBro series after appending to the 3′ end of *AtCLE* mRNA.

**Source Data Underlying Fig. 2c.** Differential fluorescence decay of 3WJ-nBro series with and without *AtCLE* mRNA.

**Source Data Underlying Fig. 2d.** Representative fluorescence images of 500 *E. coli* BL21 cells expressing 3WJ-nBro-tagged *AtCLE* mRNAs.

**Source Data Underlying Fig. 2e.** Representative fluorescence images of *N. benthamiana* leaf cells expressing 3WJ-nBro-tagged *AtCLE* mRNAs.

**Source Data Underlying Fig. 3a.** Linear regression of fluorescence intensity and mRNA content.

**Source Data Underlying Fig. 3b.** Representative image of 300 μM 3WJ-4×Bro-tagged mRNAs in vitro.

**Source Data Underlying Fig. 2c.** Quantification of the fluorescence of RNA–DFHBI-1T complexes.

**Source Data Underlying Fig. 2d.** Comparison of the fluorescence decay of 3WJ-4×Bro-tagged mRNAs within 5 h after DFHBI-1T administration.

**Source Data Underlying Fig. 4a.** Representative images of *E. coli* cells expressing three mRNAs tagged with 3WJ-4×Bro.

**Source Data Underlying Fig. 4b.** The percentage of detectable fluorescing cells to the 500 total cells expressing each 3WJ-4×Bro-tagged mRNAs.

**Source Data Underlying Fig. 4c.** Fluorescence quantification of mRNAs tagged with 3WJ-4×Bro in cells.

**Source Data Underlying Fig. 4d.** Assessment of the integrity of three mRNAs with the 3WJ-4×Bro tag in *E. coli* cells.

**Source Data Underlying Fig. 4e.** Immunoblot analysis of target proteins translated from 3WJ-4×Bro-tagged mRNAs.

**Source Data Underlying Fig. 5a.** Confocal imaging of *N. benthamiana* protoplasts expressing three 3WJ-4×Bro-tagged mRNAs.

**Source Data Underlying Fig. 5b.** Signal-to-background ratio of fluorescence in protoplasts incubated with 10 μM DFHBI-1T.

**Source Data Underlying Fig. 5c.** Representative confocal images of *N. benthamiana* leaves expressing three 3WJ-4×Bro-tagged mRNAs.

**Source Data Underlying Fig. 5e.** Detection of *mCherry-3WJ-4×Bro* mRNA and mCherry protein in *N. benthamiana* leaves.

**Source Data Underlying Fig. 5f.** Confocal microscopy detection of dynamic nuclear export process of *NtTub*α mRNA with 3WJ-4×Bro in *N. benthamiana* leaf cells at 0, 24, 48, and 72 h post infiltration.

**Source Data Underlying Fig. 6b.** Fluorescence signals of *N. benthamiana* leaves under ultraviolet and confocal microscopes.

**Source Data Underlying Fig. 7a.** Identification of T_1_ and T_2_ transgenic *A. thaliana*.

**Source Data Underlying Fig. 7c.** Expression of *NtTubα*-*3WJ-4×Bro* in different tissues of transgenic *A. thaliana*.

**Source Data Underlying Fig. 7e.** Quantitative comparison of fluorescence intensity between the 3WJ-4×Bro/DFBH-1T and GFP reporter systems.

**Source Data Underlying Fig. S4.** Ratios of fluorescent *E. coli* cells to total cells expressing *AtCLE* mRNAs tagged with different aptamers.

**Source Data Underlying Fig. S5.** Fluorescence intensities of *E. coli* cells expressing *AtCLE* mRNAs tagged with different aptamers.

**Source Data Underlying Fig. S10.** Prokaryotic expression of 3WJ-4×Bro-tagged genes.

**Source Data Underlying Fig. S12.** Quantification of fluorescence intensity in protoplasts expressing mRNAs tagged with 3WJ-4×Bro.

**Source Data Underlying Fig. S14.** Integrity detection of mRNAs tagged with 3WJ-4×Bro in *Nicotiana benthamiana* cells.
