# Supplementary material for: A protein-independent fluorescent RNA aptamer reporter system for plant genetic engineering
Source: Nat Commun. 2020 Jul 31;11:3847. doi: 10.1038/s41467-020-17497-7 (PMC7395781; doi:10.1038/s41467-020-17497-7)
Supplement: Supplementary file 4 — Source Data [file 41467_2020_17497_MOESM4_ESM.zip › Source Data/Source Data Underlying Fig. 4d .docx]

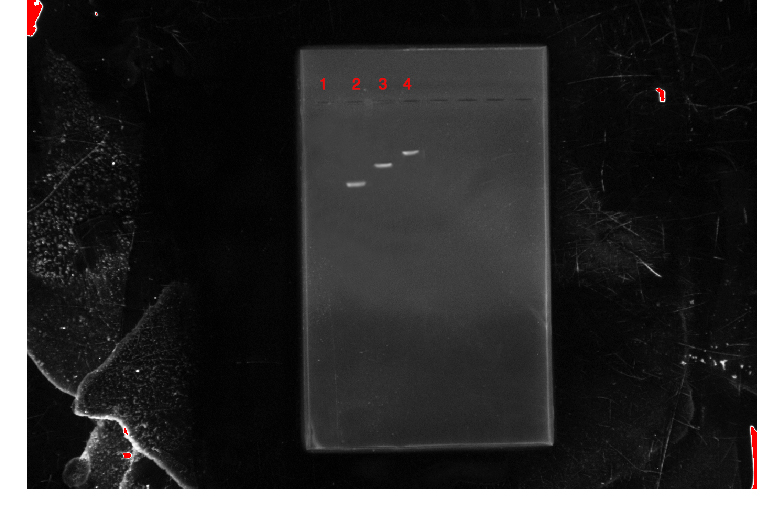


Fig. 4d Assessment of the integrity of three mRNAs with the 3WJ-4×Bro tag in E. coli cells

Lane 1, marker; Lane 2, RNA of AtCLE-3WJ-4×Bro; Lane 3, RNA of mCherry -3WJ-4×Bro; Lane 4, RNA of NtTubα-3WJ-4×Bro.
