# Supplementary material for: A protein-independent fluorescent RNA aptamer reporter system for plant genetic engineering
Source: Nat Commun. 2020 Jul 31;11:3847. doi: 10.1038/s41467-020-17497-7 (PMC7395781; doi:10.1038/s41467-020-17497-7)
Supplement: Supplementary file 4 — Source Data [file 41467_2020_17497_MOESM4_ESM.zip › Source Data/Source Data Underlying Fig. S10 .docx]

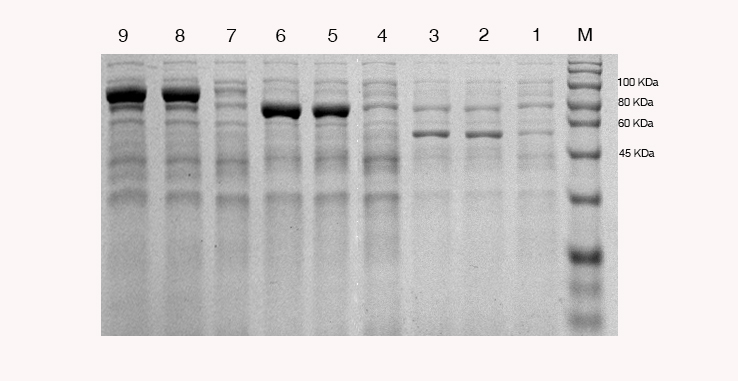


**Prokaryotic expression of 3WJ-4×Bro-tagged genes.** Lane M, protein marker; Lane 1, total proteins from *E. coli* cells without inducible expression of *AtCLE*; Lane 2, total proteins from *E. coli* cells expressing *AtCLE*; Lane 3, total proteins from *E. coli* cells expressing *AtCLE-3WJ-4×Bro*; Lane 4, total proteins from *E. coli* cells without inducible expression of *mCherry*; Lane 5, total proteins from *E. coli* cells expressing *mCherry*; Lane 6, total proteins from *E. coli* cells expressing *mCherry* -*3WJ-4×Bro*; Lane 7, total proteins from *E. coli* cells without inducible expression of *NtTubα*; Lane 8, total proteins from *E. coli* cells expressing *NtTub*α; Lane 9, total proteins from *E. coli* cells expressing *NtTubα-3WJ-4×Bro*.
