# Supplementary material for: A protein-independent fluorescent RNA aptamer reporter system for plant genetic engineering
Source: Nat Commun. 2020 Jul 31;11:3847. doi: 10.1038/s41467-020-17497-7 (PMC7395781; doi:10.1038/s41467-020-17497-7)
Supplement: Supplementary file 4 — Source Data [file 41467_2020_17497_MOESM4_ESM.zip › Source Data/Source Data Underlying Fig. S14 .docx]

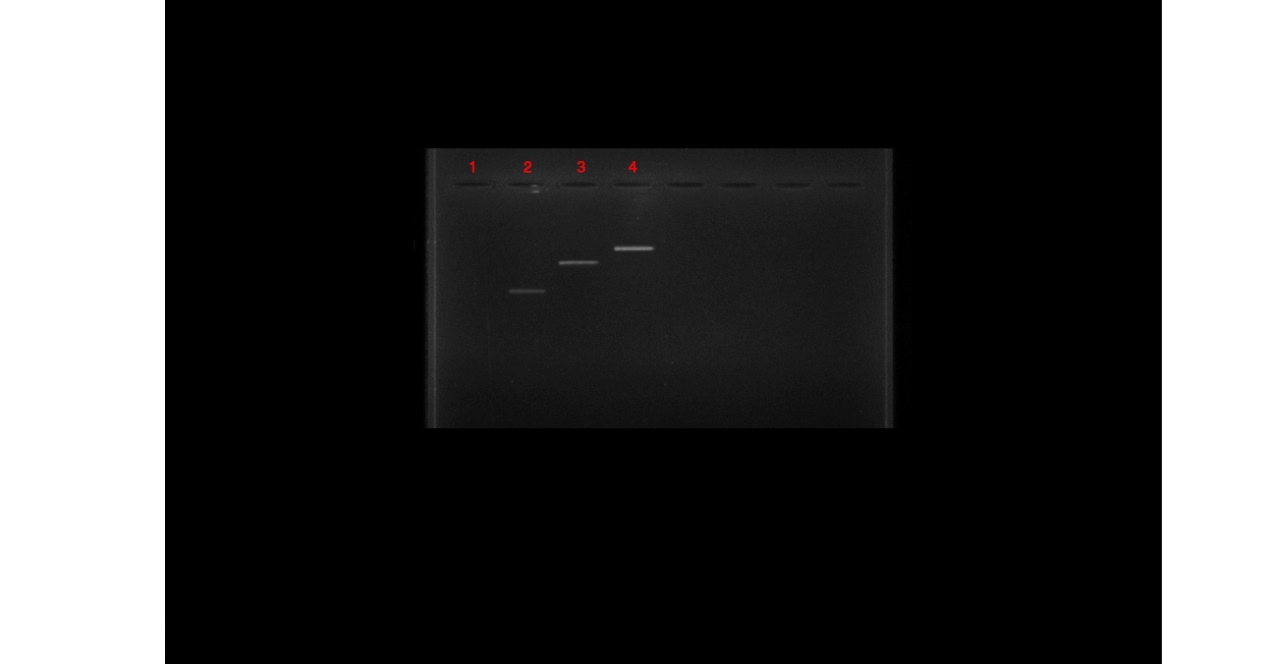


**Integrity detection of mRNAs tagged with 3WJ-4×Bro in *Nicotiana benthamiana* cells.**

Lane 1, marker; Lane 2, RNA of *AtCLE-3WJ-4×Bro*; Lane 3, RNA of *mCherry* -*3WJ-4×Bro*; Lane 4, RNA of *NtTubα-3WJ-4×Bro*.
