# Supplementary material for: A protein-independent fluorescent RNA aptamer reporter system for plant genetic engineering
Source: Nat Commun. 2020 Jul 31;11:3847. doi: 10.1038/s41467-020-17497-7 (PMC7395781; doi:10.1038/s41467-020-17497-7)
Supplement: Supplementary file 4 — Source Data [file 41467_2020_17497_MOESM4_ESM.zip › Source Data/Source Data Underying Fig. 7a and 7b/PCR identification of T1 and T2 transgenic plants .docx]

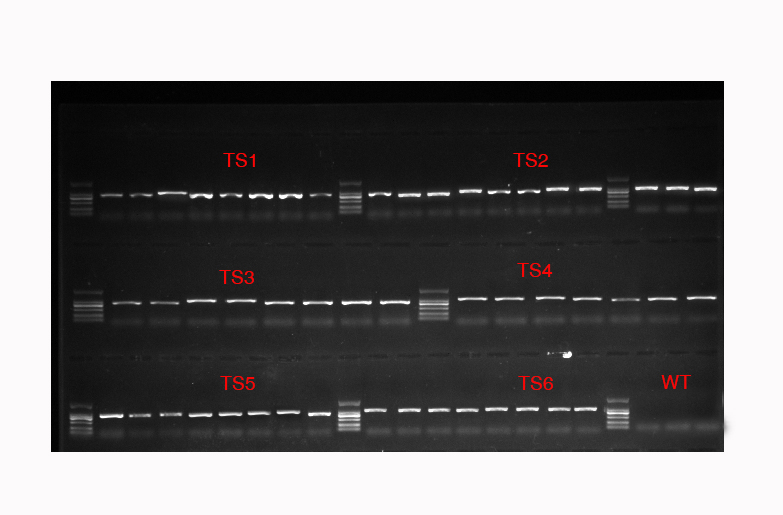

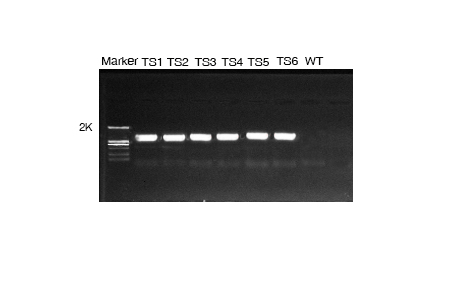
RT-PCR identification of T_1_ Transgenic Arabidopsis thaliana. TS, transgenic lines; WT, wild type; T_1_, T_2_, transgenic generations 1 and 2.

RT-PCR identification of T_2_ Transgenic Arabidopsis thaliana. TS, transgenic lines; WT, wild type; T_1_, T_2_, transgenic generations 1 and 2.
