# Supplementary figures and images for: A protein-independent fluorescent RNA aptamer reporter system for plant genetic engineering
Source: Nat Commun. 2020 Jul 31;11:3847. doi: 10.1038/s41467-020-17497-7 (PMC7395781; doi:10.1038/s41467-020-17497-7)

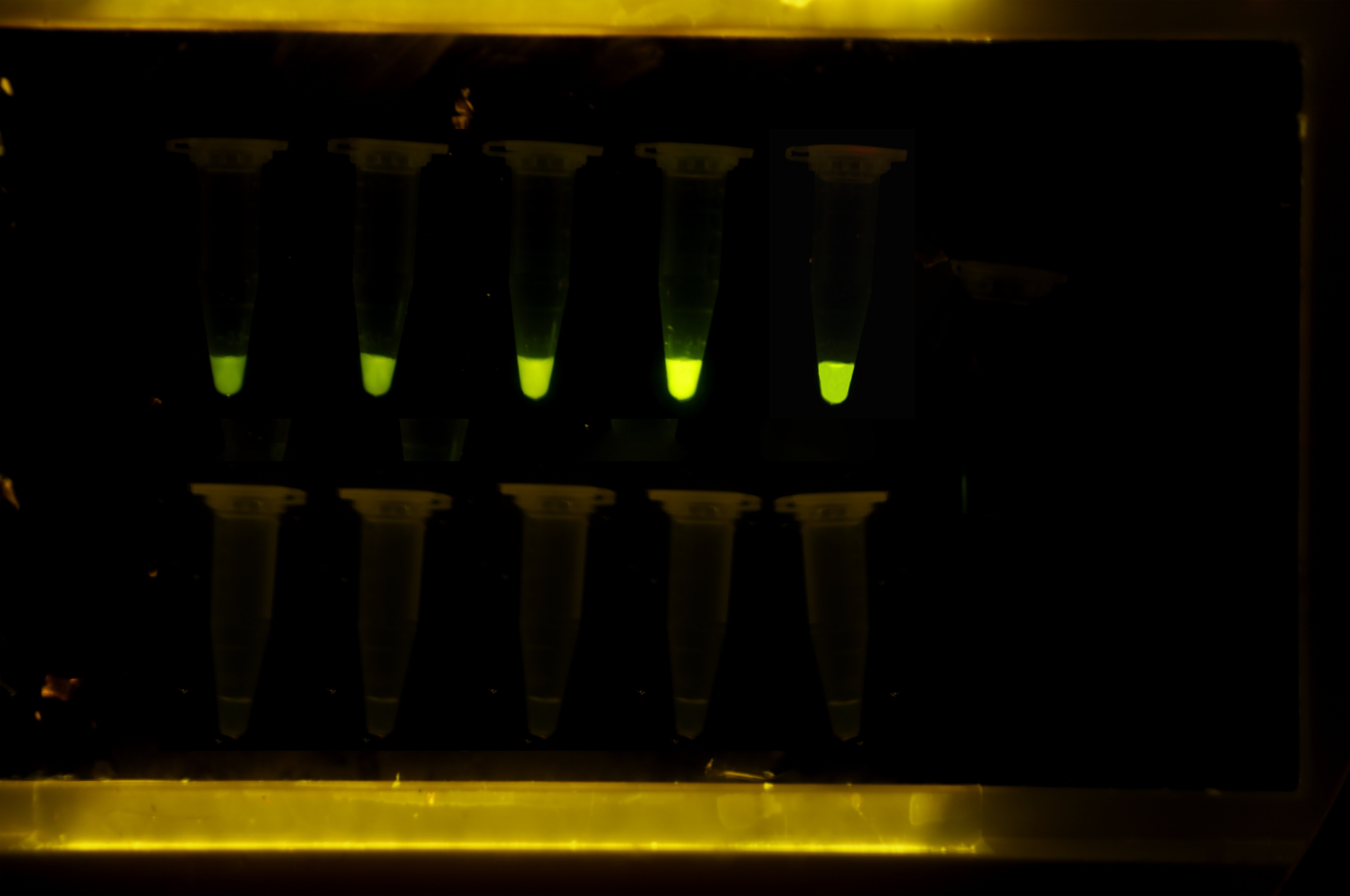

Supplement: Supplementary file 4 — Source Data [file 41467_2020_17497_MOESM4_ESM.zip › Source Data/Source Data Underlying Fig. 1b .jpg]

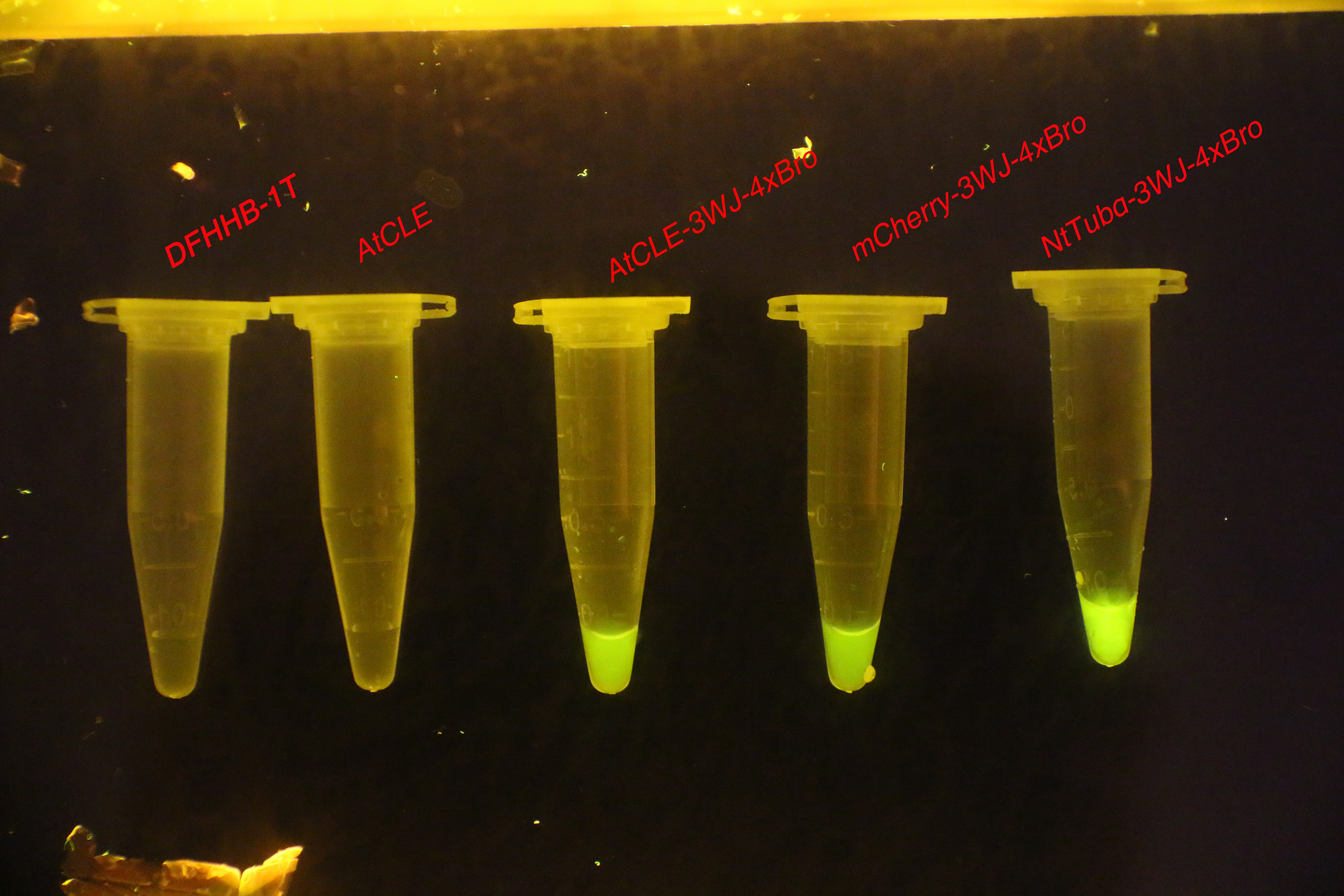

Supplement: Supplementary file 4 — Source Data [file 41467_2020_17497_MOESM4_ESM.zip › Source Data/Source Data Underlying Fig. 3b/IMG_6660.jpg]

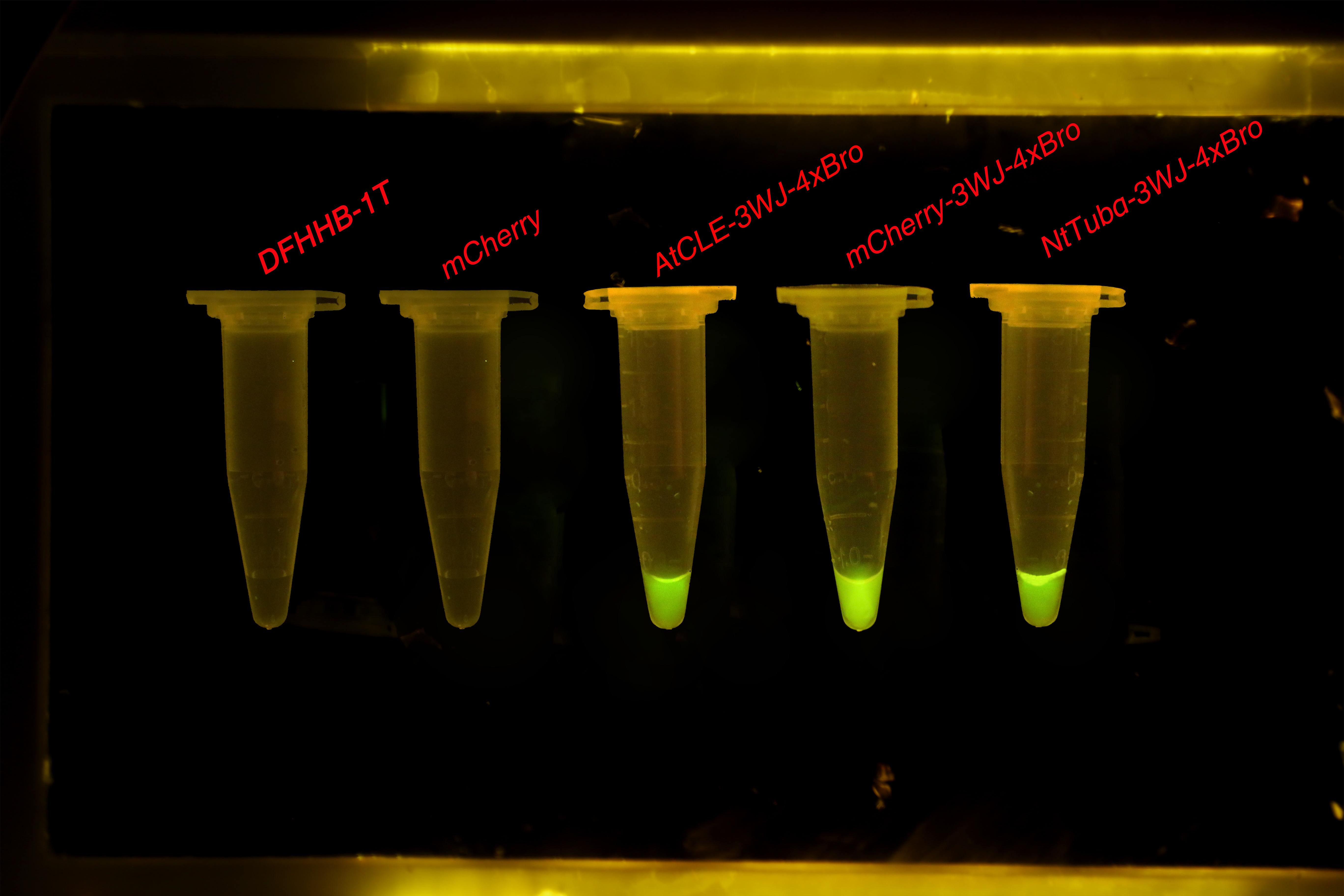

Supplement: Supplementary file 4 — Source Data [file 41467_2020_17497_MOESM4_ESM.zip › Source Data/Source Data Underlying Fig. 3b/IMG_6661.jpg]

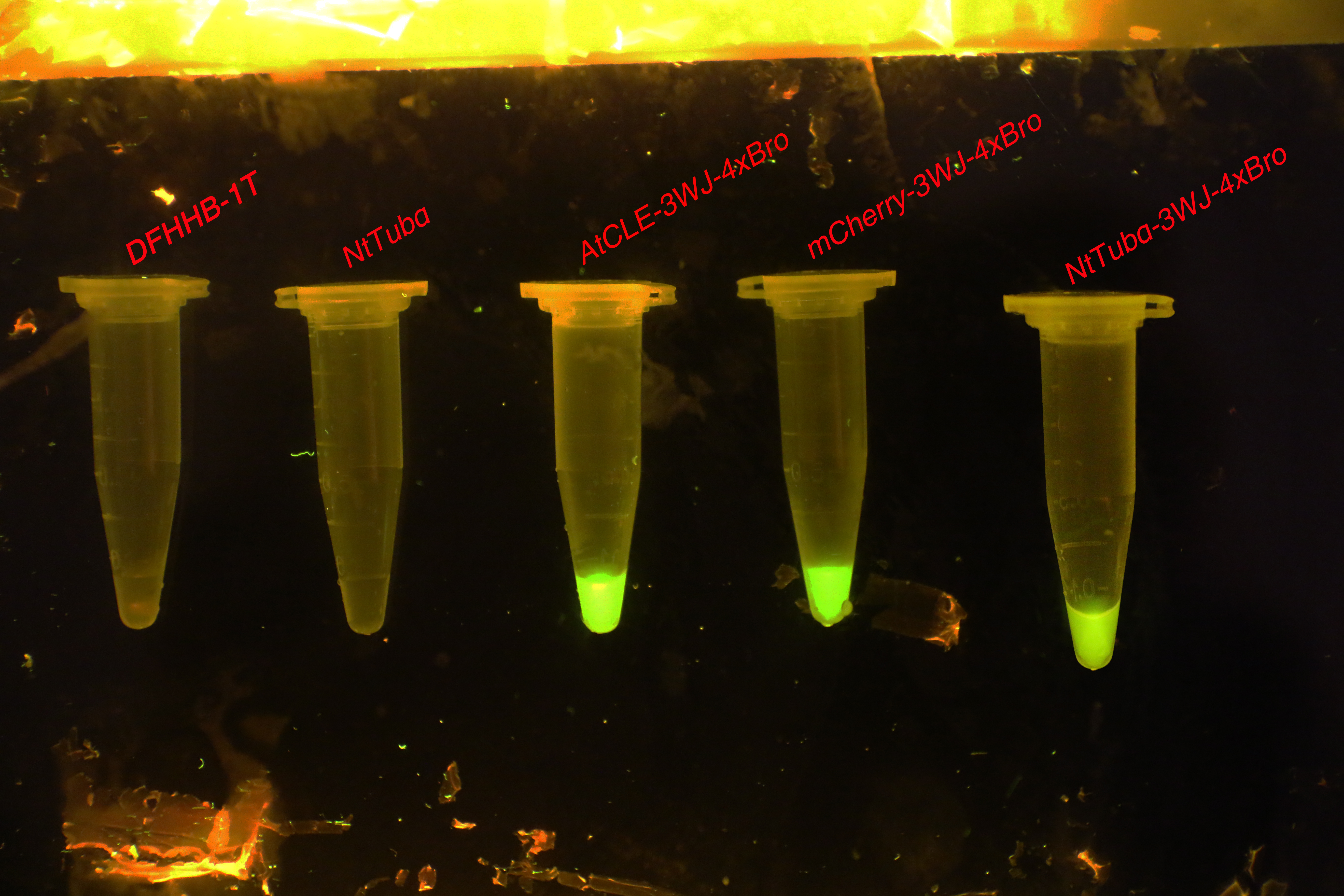

Supplement: Supplementary file 4 — Source Data [file 41467_2020_17497_MOESM4_ESM.zip › Source Data/Source Data Underlying Fig. 3b/IMG_6663.jpg]

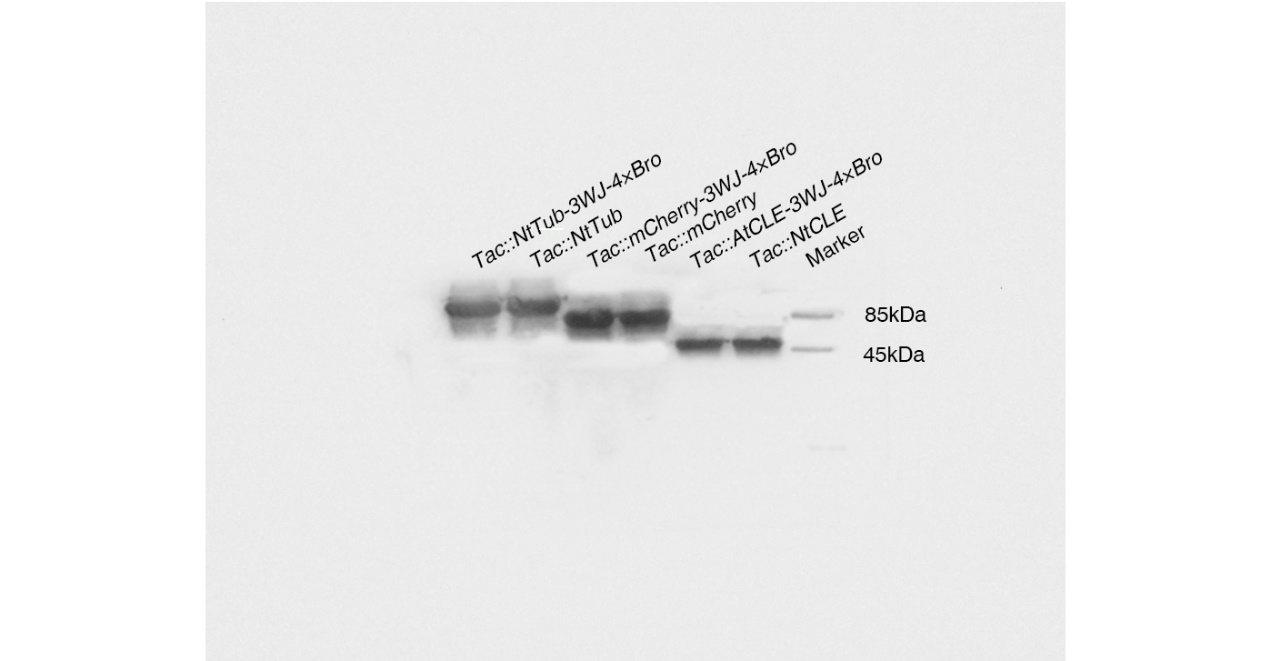


**Fig 4e** Immunoblot analysis of target proteins translated from3WJ-4×Bro-tagged mRNAs.

Supplement: Supplementary file 4 — Source Data [file 41467_2020_17497_MOESM4_ESM.zip › Source Data/Source Data Underlying Fig. 4e .docx]

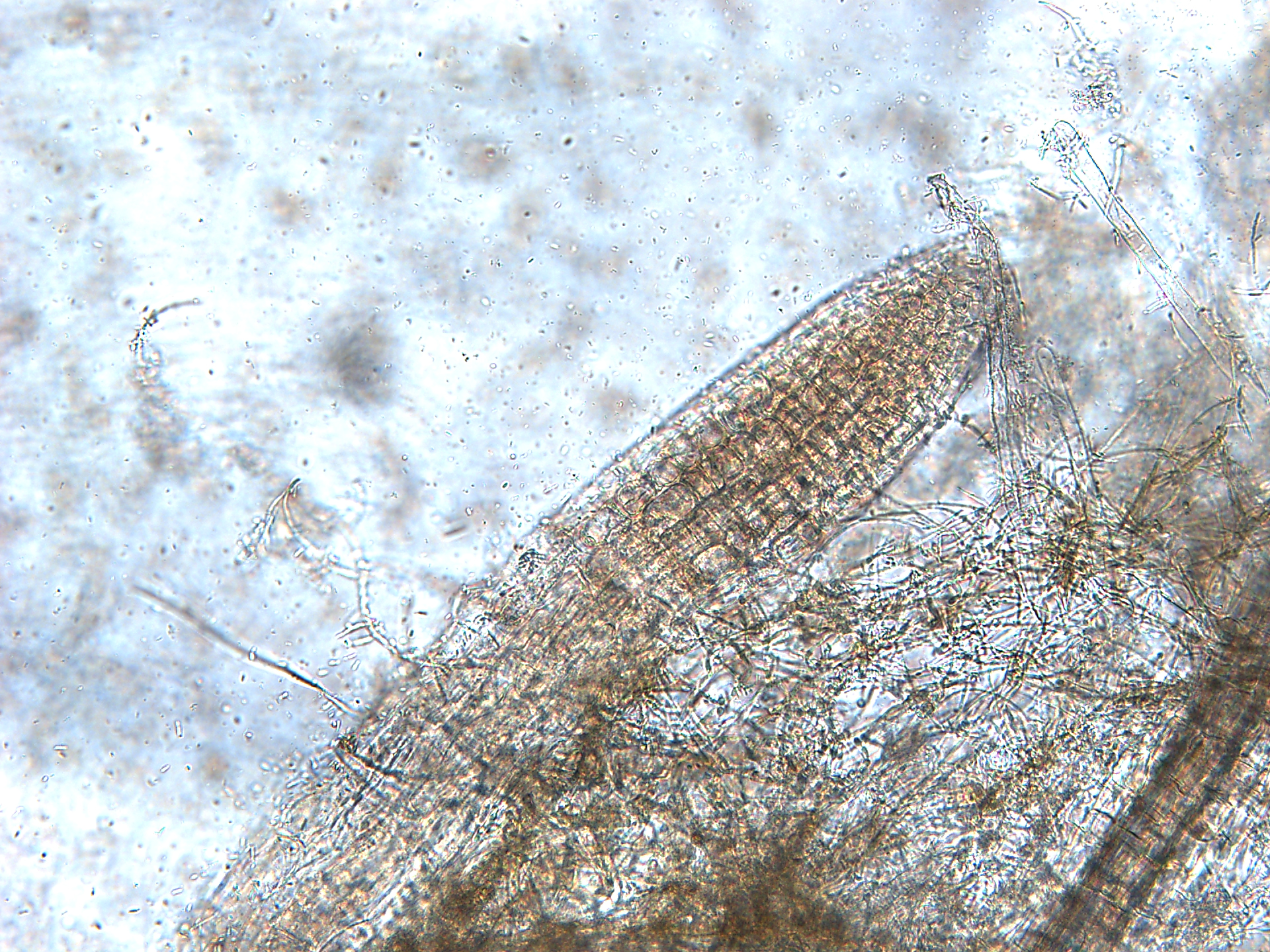

Supplement: Supplementary file 4 — Source Data [file 41467_2020_17497_MOESM4_ESM.zip › Source Data/Source Data Underying Fig. 7c/Transgene-calyptra-bright.jpg]

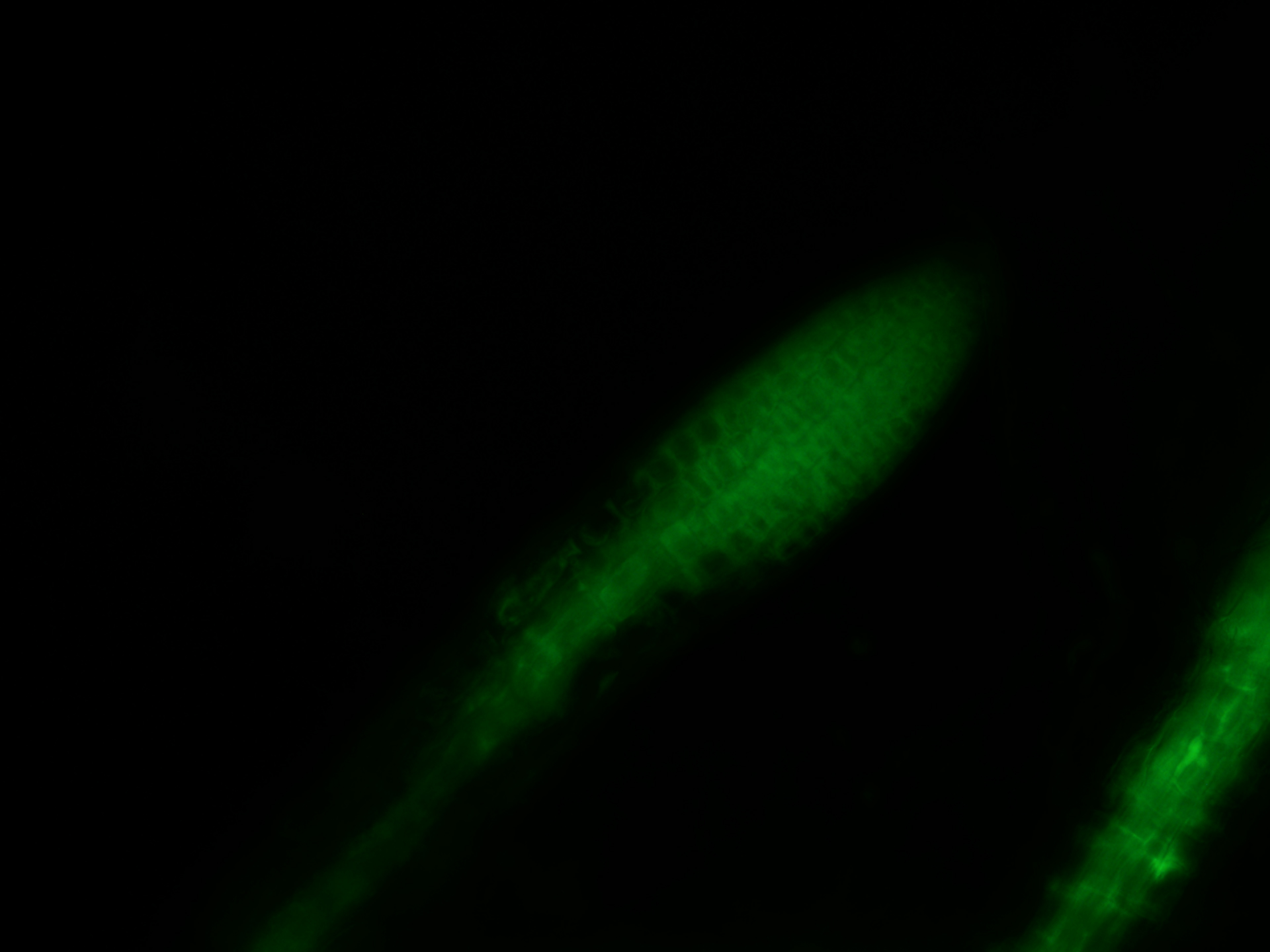

Supplement: Supplementary file 4 — Source Data [file 41467_2020_17497_MOESM4_ESM.zip › Source Data/Source Data Underying Fig. 7c/Transgene-calyptra-fluorescence.jpg]

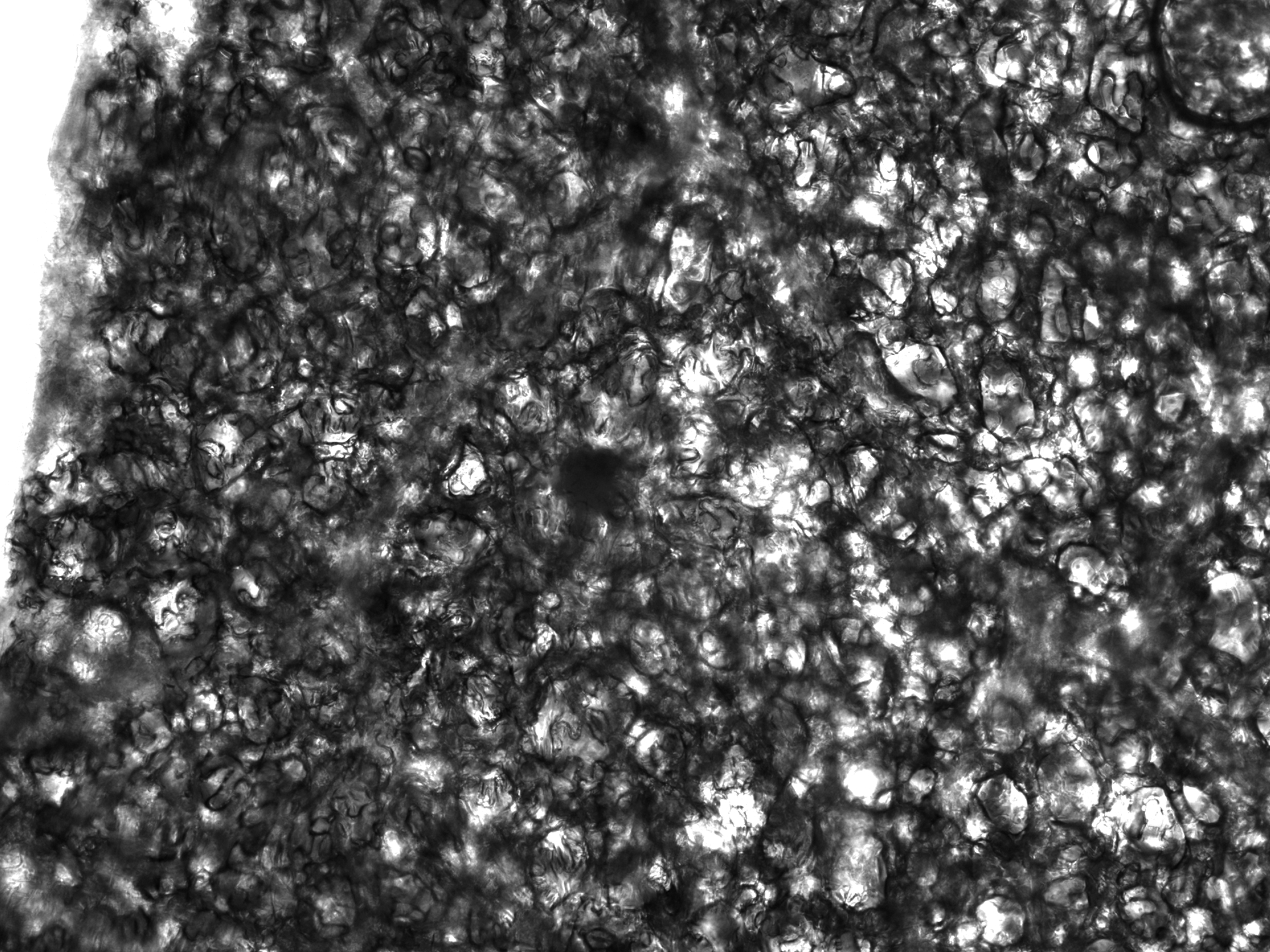

Supplement: Supplementary file 4 — Source Data [file 41467_2020_17497_MOESM4_ESM.zip › Source Data/Source Data Underying Fig. 7c/Transgene-leaf-bright .jpg]

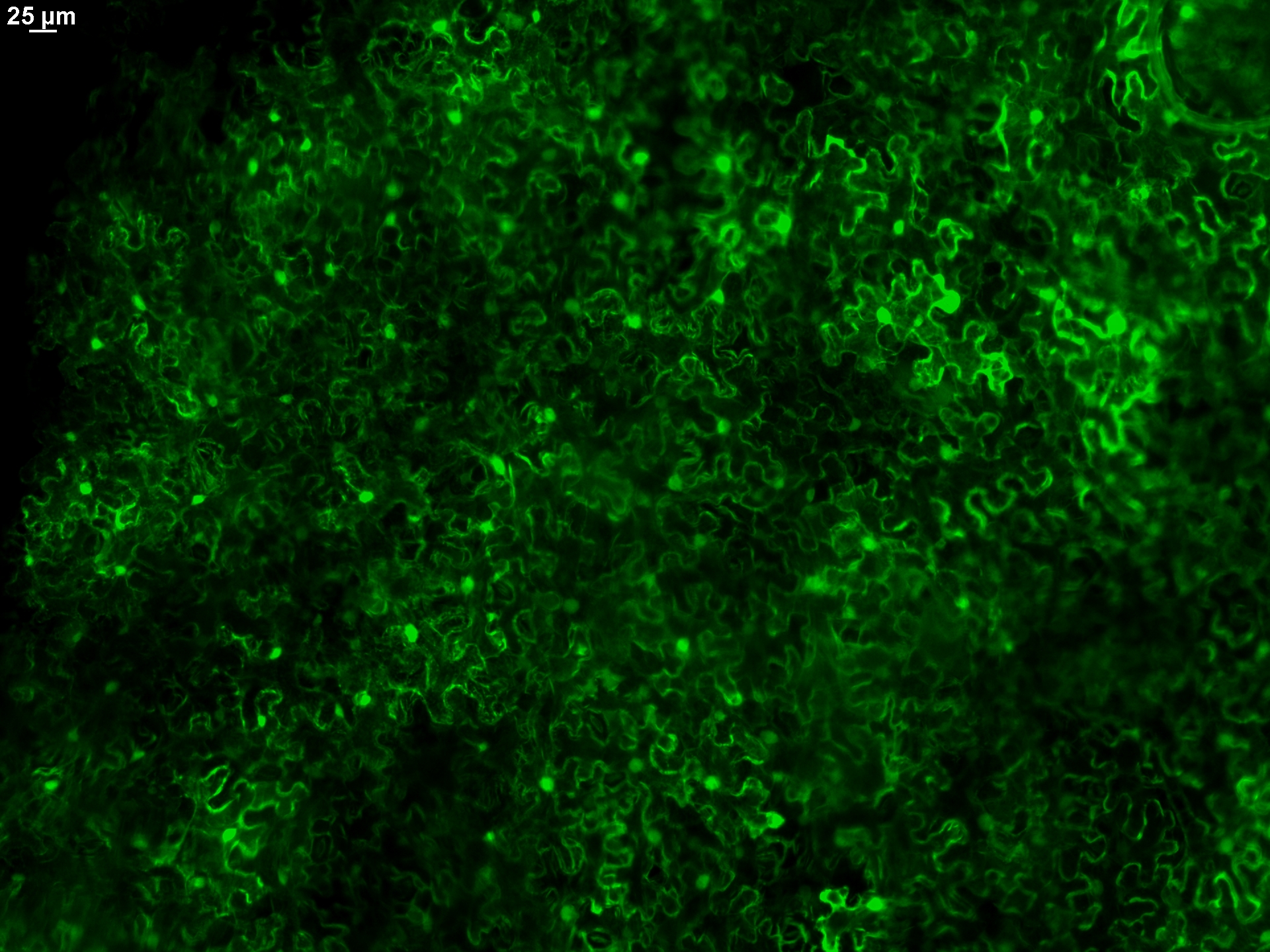

Supplement: Supplementary file 4 — Source Data [file 41467_2020_17497_MOESM4_ESM.zip › Source Data/Source Data Underying Fig. 7c/Transgene-leaf-fluorescence .jpg]

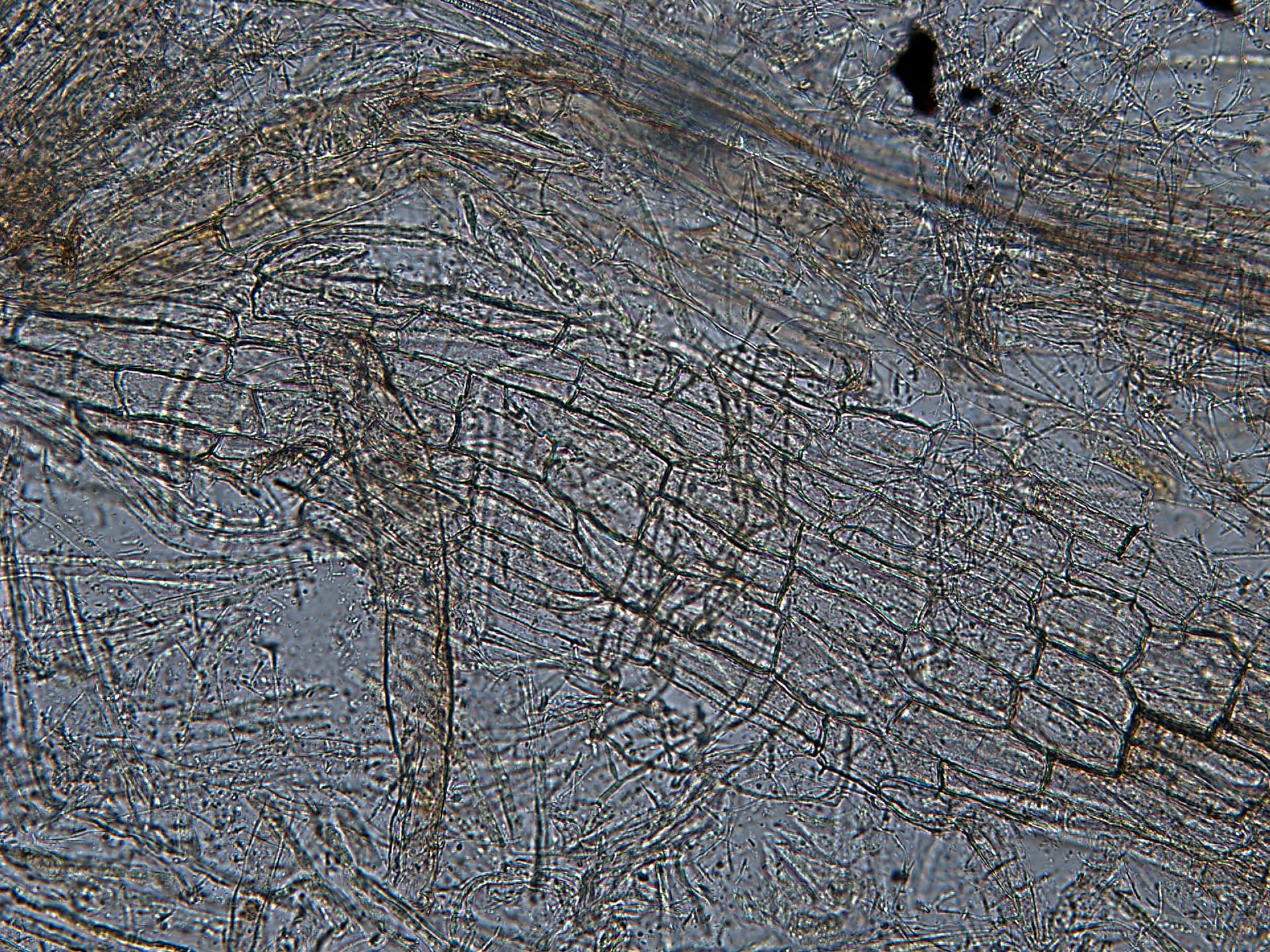

Supplement: Supplementary file 4 — Source Data [file 41467_2020_17497_MOESM4_ESM.zip › Source Data/Source Data Underying Fig. 7c/Transgene-root-bright.jpg]

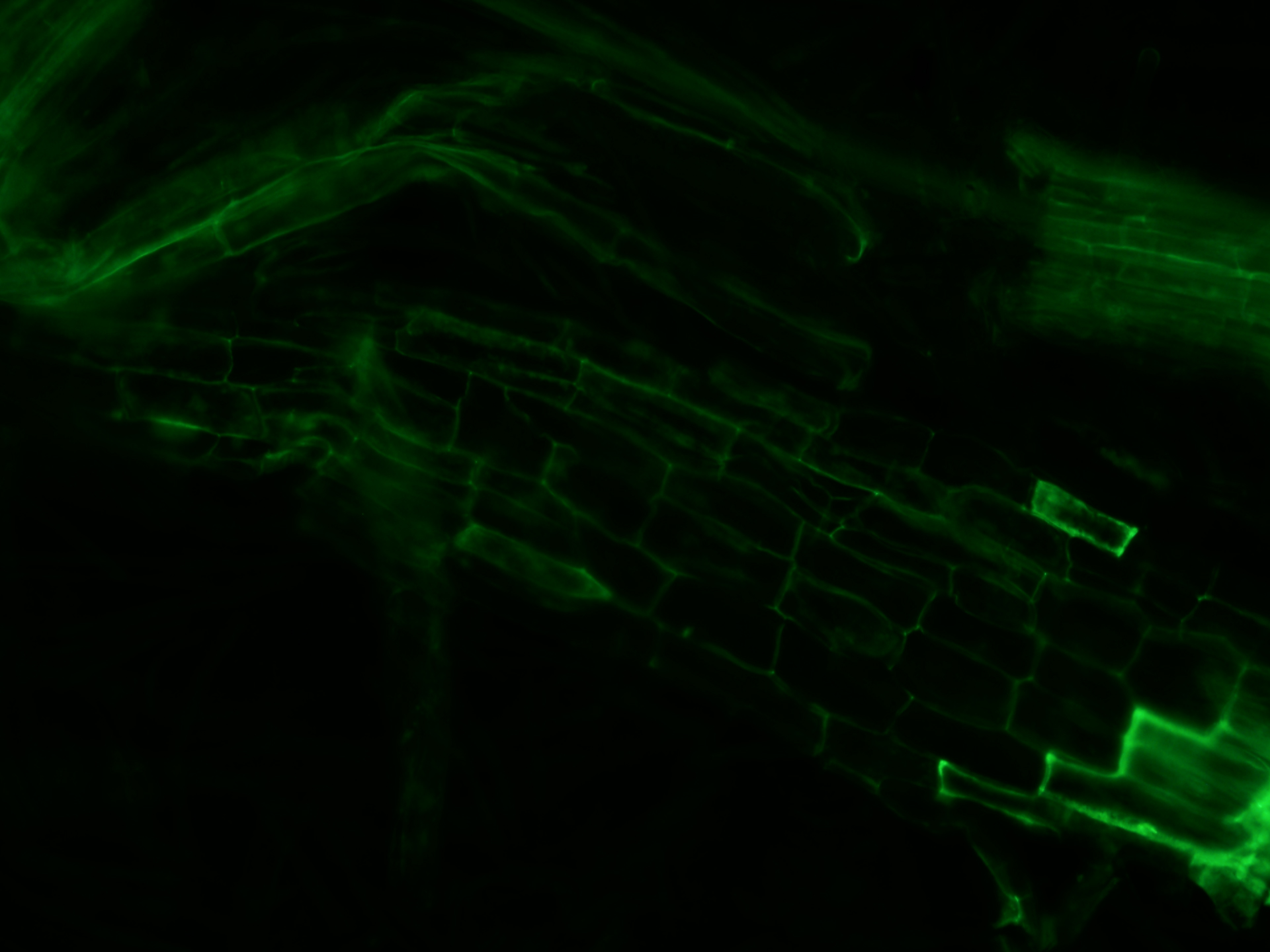

Supplement: Supplementary file 4 — Source Data [file 41467_2020_17497_MOESM4_ESM.zip › Source Data/Source Data Underying Fig. 7c/Transgene-root-fluorescence.jpg]

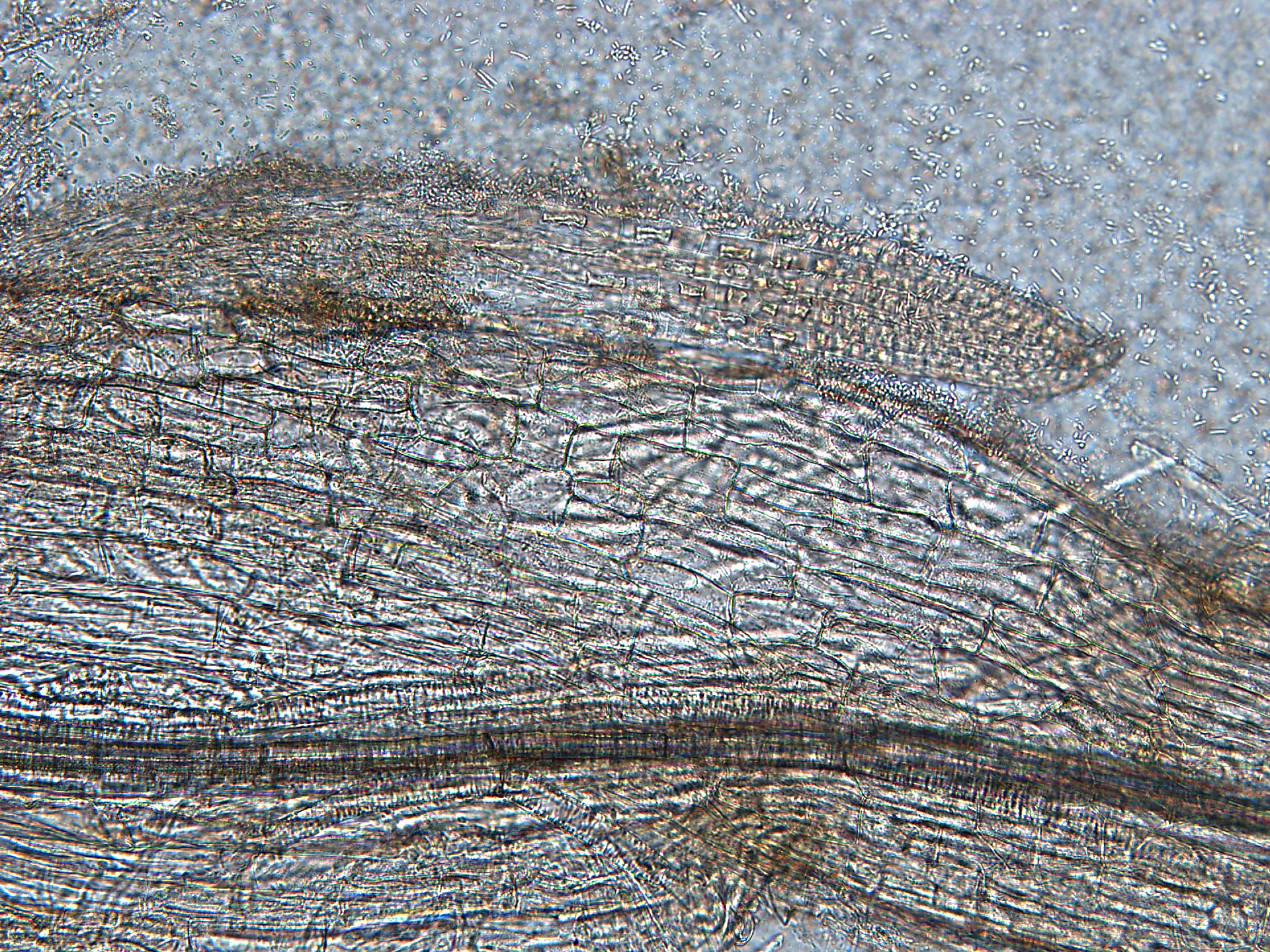

Supplement: Supplementary file 4 — Source Data [file 41467_2020_17497_MOESM4_ESM.zip › Source Data/Source Data Underying Fig. 7c/WT-calyptra-bright.jpg]

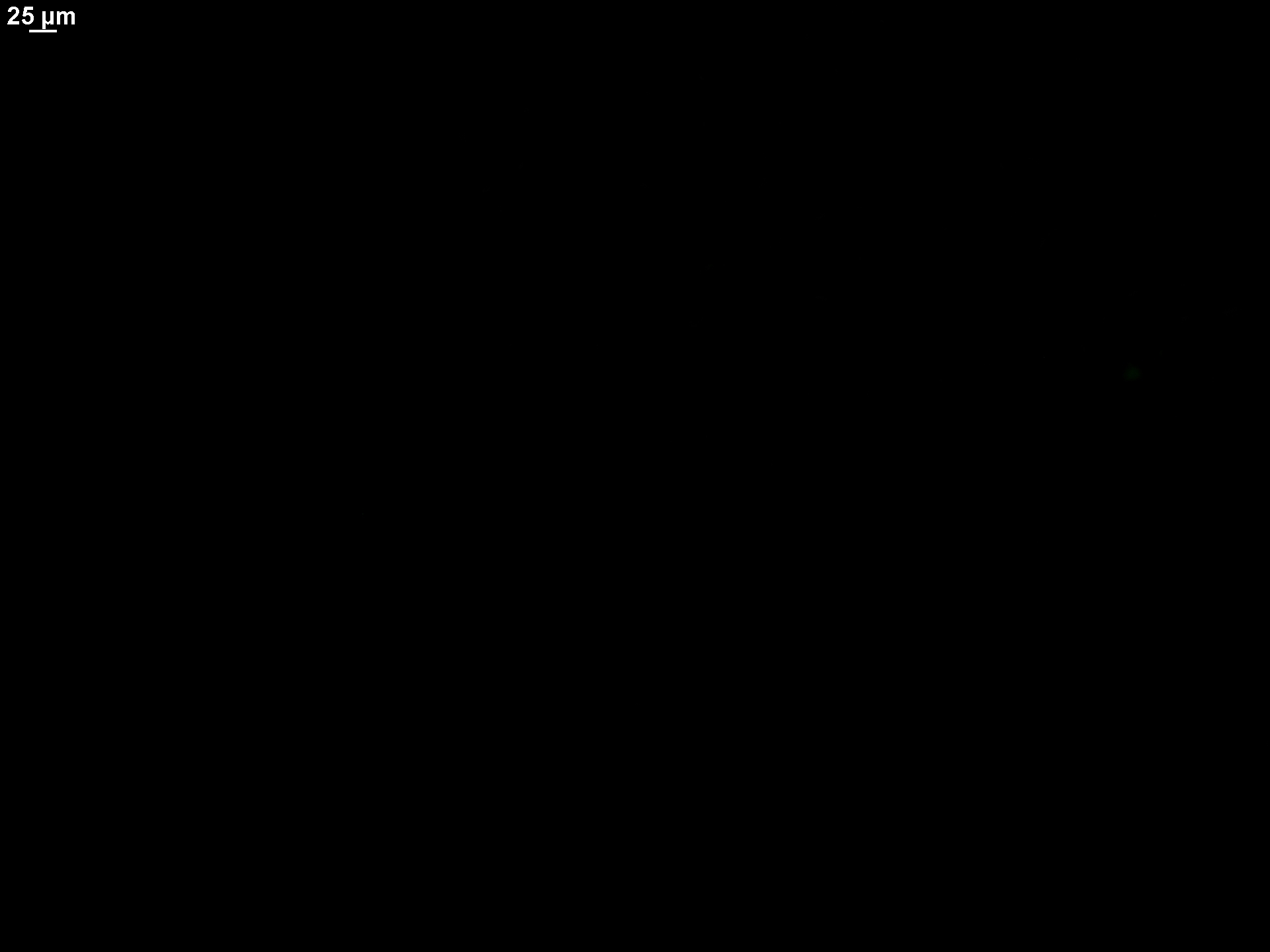

Supplement: Supplementary file 4 — Source Data [file 41467_2020_17497_MOESM4_ESM.zip › Source Data/Source Data Underying Fig. 7c/WT-calyptra-fluorescence .jpg]

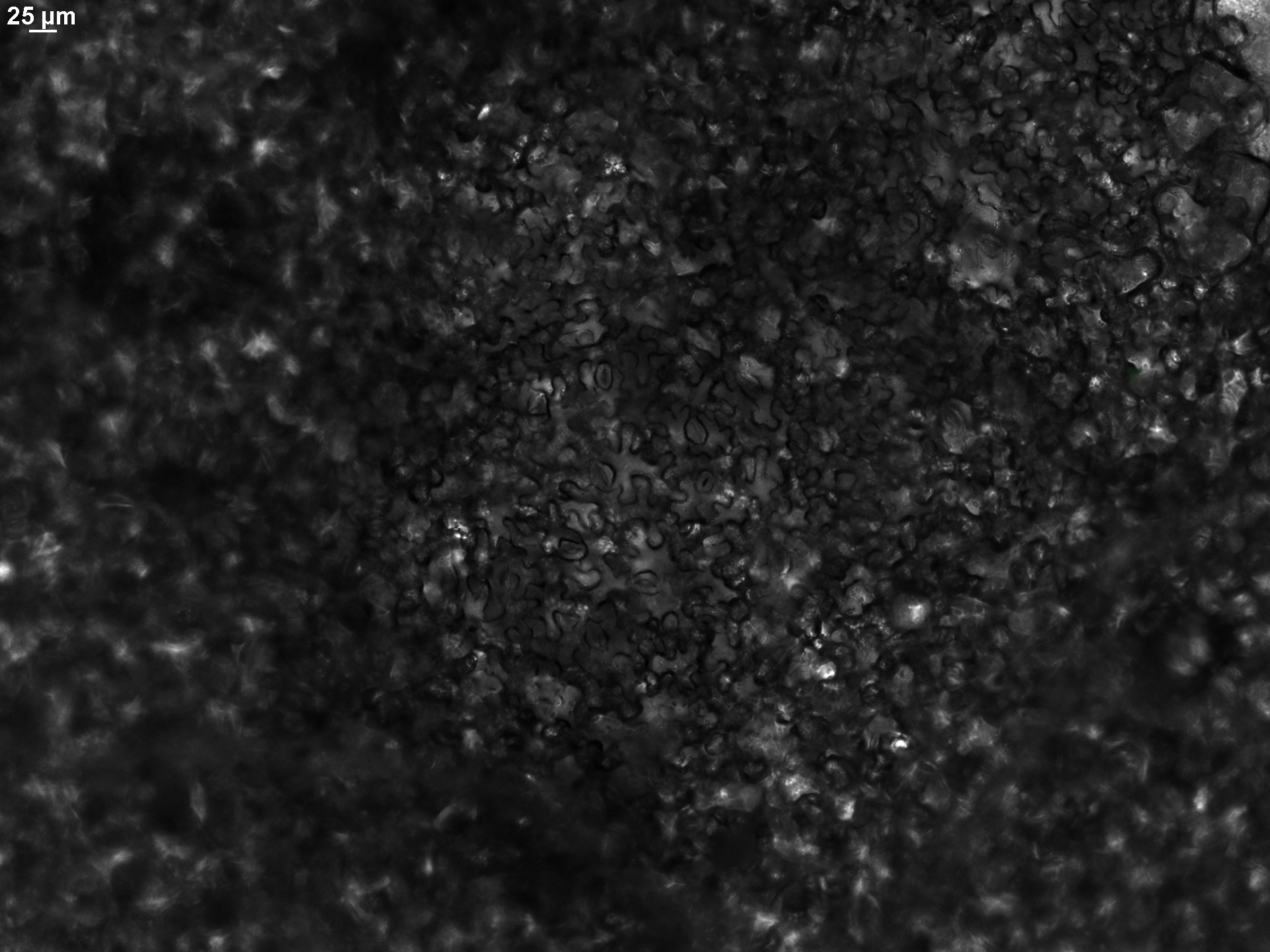

Supplement: Supplementary file 4 — Source Data [file 41467_2020_17497_MOESM4_ESM.zip › Source Data/Source Data Underying Fig. 7c/WT-leaf-bright .jpg]

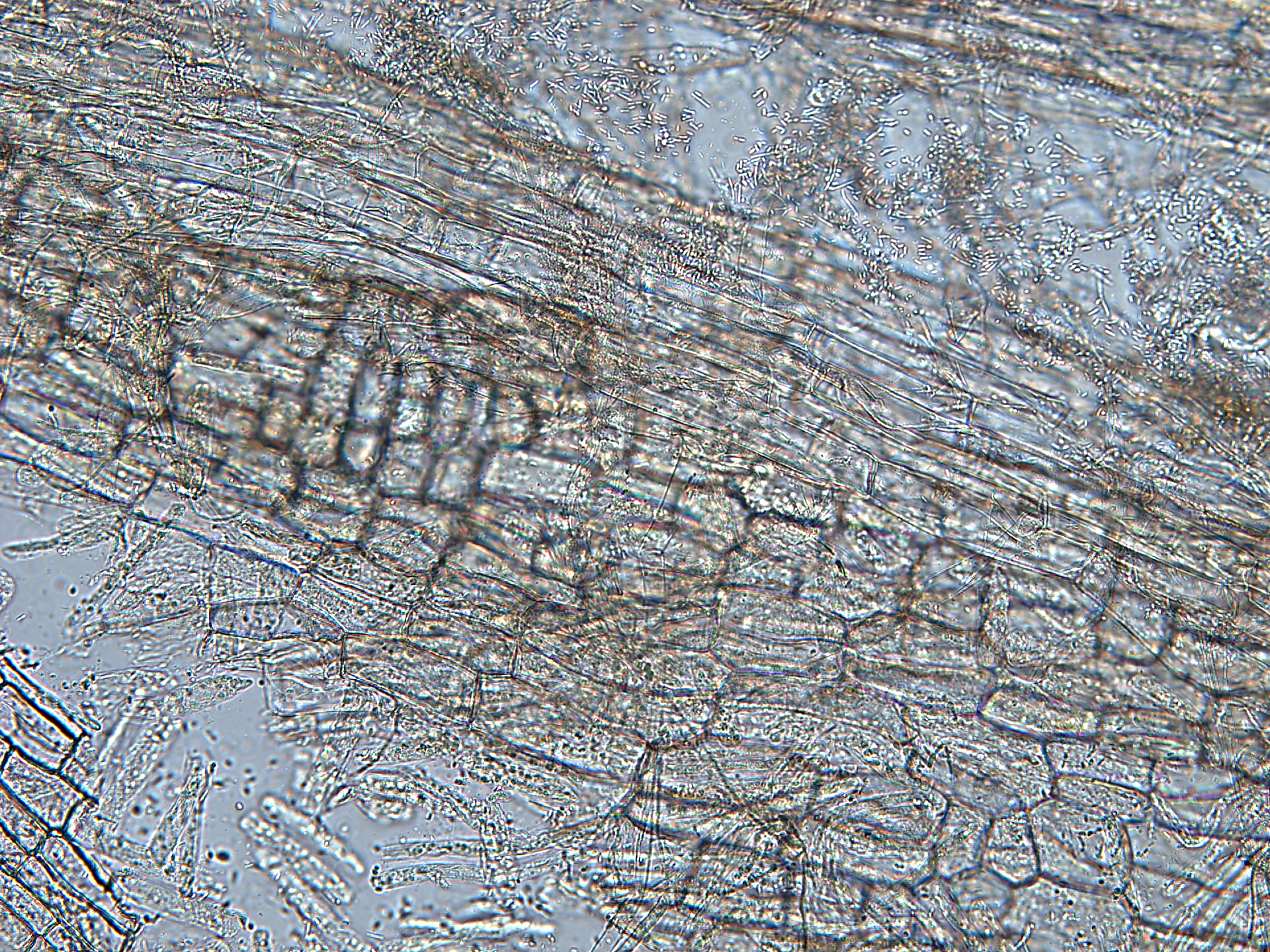

Supplement: Supplementary file 4 — Source Data [file 41467_2020_17497_MOESM4_ESM.zip › Source Data/Source Data Underying Fig. 7c/WT-root-bright.jpg]
